# Accelerating language emergence by functional pressures

**DOI:** 10.1371/journal.pone.0295748

**Published:** 2023-12-14

**Authors:** Kasun Vithanage, Rukshan Wijesinghe, Alex Xavier, Dumindu Tissera, Sanath Jayasena, Subha Fernando

**Affiliations:** 1 CODEGEN QBITS Lab, University of Moratuwa, Moratuwa, Sri Lanka; 2 Department of Computational Mathematics, Faculty of Information Technology, University of Moratuwa, Moratuwa, Sri Lanka; Shanghai Maritime University, CHINA

## Abstract

In language emergence, neural agents acquire communication skills by interacting with one another and the environment. Through these interactions, agents learn to connect or ground their observations to the messages they utter, forming a shared consensus about the meaning of the messages. Such connections form what we refer to as a grounding map. However, these maps can often be complicated, unstructured, and contain redundant connections. In this paper, we introduce two novel functional pressures, modeled as differentiable auxiliary losses, to simplify and structure the grounding maps. The first pressure enforces compositionality via topological similarity, which has been previously discussed but has not been modeled or utilized as a differentiable auxiliary loss. The second functional pressure, which is conceptually novel, imposes sparsity in the grounding map by pruning weaker connections while strengthening the stronger ones. We conduct experiments in multiple value-attribute environments with varying communication channels. Our methods achieve improved out-of-domain regularization and rapid convergence over baseline approaches. Furthermore, introduced functional pressures are robust to the changes in experimental conditions and able to operate with minimum training data. We note that functional pressures cause simpler and more structured emergent languages showing distinct characteristics depending on the functional pressure employed. Enhancing grounding map sparsity yields the best performance and the languages with the most compressible grammar. In summary, our novel functional pressures, focusing on compositionality and sparse groundings, expedite the development of simpler, more structured languages while enhancing their generalization capabilities. Exploring alternative types of functional pressures and combining them in agent training may be beneficial in the ongoing quest for improved emergent languages.

## 1. Introduction

Language emergence involves training neural agents to autonomously forge their own language by interacting with peers and their surroundings [1]. The potential scope of such techniques ranges from multi-agent collaboration [2] to fine-tuning large language models [3]. Nonetheless, emergent languages can be complex, lack clear structure [4], and frequently diverge from salient characteristics found in natural languages [5–7]. Natural languages, which are structured and generalizing, exhibit important properties like compositionality. Hence, existing studies have put effort into investigating the mechanisms of generating emergent languages that are qualitatively more analogous to natural languages and interpretable [7, 8].

Iterated learning [9], cultural transmission [10], architectural evolution of agents [11], and curriculum learning [12] are all such strategies that can enhance emergent languages in diverse ways. However, such approaches involve managing multiple generations of agents or agent replacements. Alternatively, input data distribution can be altered to produce functional pressures that guide agents to emerge languages with expected characteristics. Instead of manipulating input data, functional pressures can also be modeled as auxiliary objectives or loss components. Such losses or objectives can be optimized alongside the primary loss associated with the non-linguistic objective without much additional effort. Prior research has investigated both types of pressures and shows promising results [13].

In this paper, we present two novel functional pressures modeled as auxiliary losses to enhance the compositionality and sparsity of the grounding map. Previous works have formulated the principle of least effort similarly [7, 13]. However, the principles behind the least effort pressure and our functional pressures are different, and investigating other types of pressures, which are principally diverse, could always lead to exciting outcomes. Our methods are not based on evolutionary or iterative approaches [9, 11], and our agents complete the learning process within a single generation.

We represent compositionality, a concept that has been discussed in many existing studies [14], by evaluating the topological similarity between inputs and agent messages. Existing formulations of topological similarity are not used as differentiable auxiliary losses for optimization [4, 15, 16]. However, our work presents topological similarity in a differentiable format suitable for mini-batch-based optimization. Other differentiable representations for compositionality exist. Nevertheless, such approaches require training a separate model for estimation [17]. In contrast, our formulation is straightforward and can be estimated within a single generation of agents without training additional models. To the best of our knowledge, this is the first instance in language emergence literature where topological similarity has been directly optimized as a functional pressure in a differentiable format.

As the language evolves, it is crucial that a robust yet limited set of connections forms between fundamental concepts and messages. An excessive number of connections can threaten the early stages of language development; for instance, generating multiple synonyms at the outset of training can unnecessarily consume the available set of messages. Smaller vocabularies are easier to learn than larger ones containing redundant mappings. In summary, agents should respect the principle of parsimony when forging their languages. We introduce our second functional pressure to make the grounding maps sparse while keeping the performance in the primary objective intact. Our notion of sparsity differs from [2, 8], where they are interested in chronological or inter-agent communication sparsity, while we concern about sparsity in the grounding map.

We conduct multiple experiments by systematically varying key factors, including input data, communication channel characteristics, the percentage of training data, batch size, and learning rate. These comparisons reveal substantial advantages in generalization and convergence speed compared to the existing approaches. Our methods yield improvements even when the training data is only 10% of the total data set size and shows better out-of-domain generalization than the existing methods. Notably, the gains produced by our novel functional pressures are robust to changes in batch sizes and learning rates. Additionally, analyzing agent messages shows that our approaches emerge languages with more compact syntactical features, such as displaying shorter grammar description and data description lengths [18]. The emergent languages show unique characteristics based on the functional pressure utilized during the training. Our results indicate that grounding sparsity, which produces the most compressible grammar, is the most beneficial of the two introduced functional pressures in terms of performance.

## 2. Related work

Many experimental setups in language emergence studies draw inspiration from Lewis signaling games [19]. While many experiments share such a common paradigm, they can vary significantly regarding the environment, input data, agent objectives, and agent architecture. Reconstruction games [20, 21] and discrimination games [1, 9, 15, 22] are two of the most frequent variations within this domain. Both games involve two agent roles, Sender and Listener, and use a non-linguistic objective to train the agents.

There is a wide range of diversity when considering the types of environments used in language emergence experiments. Some works employ simulated environments where agents perform objectives related to navigation, including both 2-D [8, 23–25] and 3-D simulated environments [26], which may provide insight into real-world multi-agent cooperation. The use of abstract symbolic data [20] is an alternative, which provides a more simplified environment for analyzing and quantifying the properties of emergent languages.

Recently, emergent communication has been applied with large pre-trained language models as a fine-tuning step, improving translation results [3]. To facilitate agent communication, various modalities and control mechanisms have been employed. These encompass visual communication [27], discrete communication [20], continuous communication [24], and quantization-type communication channels [28]. Some recent research also explores the control of communication channels to determine when and with whom to communicate [2].

These diverse experiments provide empirical insight into the dynamics of language emergence. According to empirical observations, language and its structure are influenced by many factors, namely, the agent architecture [1, 20, 21, 29], nature of the input data [13, 15], size, heterogeneity, and connectivity of the agent population [30–33], the characteristics of the inter-agent communication channel [34] and memory capacity of agents [20, 35].

Training with a loss that solely prioritizes gameplay leads to emerging languages that remain indecipherable to humans and highly complex [27]. In the absence of biases, priors or extra supervision, agents can interpret observations in diverse ways, resulting in languages prone to inefficiency and potential violations of specific properties like Zipf’s law of abbreviation [6, 7], lacking meaningful word boundaries in relation to Harris’s Articulation Scheme [5] and not generalizing linguistic knowledge to out-of-training-distribution examples [36].

Adding certain biases to the training process causes agents to develop languages with specific desired properties or to expedite the learning process [37]. These biases may be applied through various approaches [38] and yield unique outcomes. One such approach is penalizing longer messages or incentivizing shorter ones [7, 13]. Such penalizing can make the system resilient to anti-efficient encoding [7]. Adding biases has proven to facilitate the development of human decipherable emergent communications, which are otherwise not understandable to humans [27].

Incorporating additional biases has demonstrated the potential to render communications more structured and interpretable by humans [7, 15]. Another straightforward approach is to use supervised pre-training with a human language [39]. After initializing groundings through supervised pre-training, the second stage of referential gameplay can generalize the learned groundings. In our experiments, we do not use explicit linguistic supervision, although it can be incorporated into the subsequent steps of a multi-stage learning setup, as mentioned above.

In iterated learning [40], successive generations of learners acquire language skills from their predecessors, resulting in the emergence of compositionality in the language [41, 42]. Another approach to promote compositionality involves periodically replacing agents within the agent population [10, 43]. Such languages exhibit improved generalization [10] and are more easily acquired by new learners [43]. Furthermore, curriculum learning can guide agents in developing compositional and symmetrical languages [12]. Prior works have shown that architectural and cultural co-evolution within a population of agents yields increased topographic similarity [11]. Using a diagnostic classifier during agent training has shown to make the agent compositional [44].

## 3. Methodology

We employ a modified version of the Lewis Signaling game involving two players [19], a setup commonly used in language emergence studies. These players are referred to as the Speaker and the Listener. The environment can exist in various states, only discernible by the Speaker, who possesses a set of signals that it transmits to the Listener. However, the Listener, unable to directly perceive the current environmental state, must rely on the Speaker’s signal to determine the appropriate action. For each state, there exists a unique correct action, and both players have a preference for the Listener to take the correct action. In our scenario, a single input is sufficient to define the current environmental state entirely, and the Listener’s task is to reconstruct this input by observing the Speaker’s signals or messages (refer S1 Appendix for a more intuitive explanation).

In our baseline experiments, we parameterize our players or agents using neural networks [45] (refer S2 Appendix for more information). We then establish a reward system based on the quality of the Listener’s reconstruction and proceed to train both agents using backpropagation. In the ideal scenario, throughout the training process, both agents should converge towards a signaling system [19] that works for all inputs.

We then apply our functional pressures to the baseline experiment and investigate how adding functional pressures affects the generalization, learning speed, and quality of the emerged languages. We use two functional pressures in this study, where the first enhances the compositionality of the emerging languages while the second imposes sparsity in the grounding map. Additionally, we compare our functional pressures against an instance where weight decay is added to the baseline experiments.

We have devised two differentiable metrics to effectively represent both the compositionality and grounding sparsity, where the metric for compositionality is based on topological similarity. We apply these differentiable metrics as auxiliary losses to guide the agents during training. We assess the extent of generalization and the rapidity of convergence of agents guided by our metrics, comparing their performance with agents that do not adopt such principles. Furthermore, we investigate the unique characteristics caused in emergent languages by each of the two functional pressures in a later section. We use data sampled from a value-attribute dataset as inputs. The signals the Speaker sends are discrete integer strings with a consistent length.

The primary objective of our game setup is the reconstruction of the input. We incorporate functional pressures into our experiments by integrating the developed auxiliary losses into the overall loss function. The losses represent either the topological similarity $L_{t}$ or the grounding sparsity $L_{s}$. The final loss $L_{f}$ (refer to Eq 1) is, therefore, a weighted sum of the reconstruction loss and one of the two auxiliary losses, which acts as guidance for agents in acquiring linguistic properties of interest.

$$\begin{array}{c} L_{f}=L\left(x,x\prime \right)+kC_{t}L_{t}+\left(1-k\right)C_{s}L_{s},\;\;k\in \left\{0,1\right\} \end{array}$$ (1)

### 3.1 Value-attribute dataset and discrete messages

**Fig 1:**
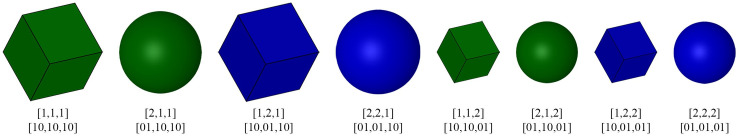
A value attribute environment has three attributes(Shape, Color, and Size), each with two possible values. Hence, there are 8 distinct objects within this environment.

Value-attribute dataset is a frequently used abstract dataset [9, 20, 21] in language emergence research. Consider the objects depicted in Fig 1. There are eight unique abstract objects in the image, each of which can be perfectly described by its Color, Shape, and Size, which we identify as attributes of the objects. Each attribute has a value; for instance, an object’s color can be green or blue. A value-attribute dataset is an abstract representation of a collection of such objects. We represent each object as a string of integers of length *A*. Each position in the string corresponds to a particular attribute of the object. Integers vary between a minimum of 1 and a predetermined maximum of *V*, so there are *V* possible values for a single attribute.

Consequently, a value-attribute dataset *X*(*V*, *A*), can be represented by two integers $V,A\in Z^{+}$. Any data point $x_{i}\in X$ represents an object comprised of *A* attributes and *V* potential values for each attribute. Therefore, the resulting dataset will contain *V*^*A*^ unique data points (refer Eqs 2 and 3). We use one-hot encoding to represent values, making *x*_*i*_ a binary vector with a size of ∣ *x*_*i*_ ∣ = *VA*. Changing the number of values *V* and the number of attributes *A* yields datasets of arbitrary size and composition.

$$\begin{array}{c} V=\{c:c\in {\left\{0,1\right\}}^{V},\sum^{V}c_{i}=1\} \end{array}$$ (2)

$$\begin{array}{c} X=\{(v_{1},\cdots ,v_{j},\cdots ,v_{A}):v_{j}\in V,1\leq j\leq A\} \end{array}$$ (3)

Messages m∈M of length *T* consists of *T* discrete symbols, is produced recurrently by sampling from a vocabulary of integers of size *S* represented as one hot encoded vectors (refer Eqs 4 and 5). Therefore, *S*^*T*^ unique messages are available for the agents, and we refer to this quantity as channel capacity. Moreover, a single message has a size of ∣ *x*_*i*_ ∣ = *ST*. We refer to tuple *M*(*S*, *T*) as the channel configuration, where the vocabulary size is *S* and the message length is *T*. We use a discrete communication channel and one-hot encoded symbols as it is the most common practice [1, 20, 23, 46].

$$\begin{array}{c} S=\{c:c\in {\left\{0,1\right\}}^{S},\sum^{S}c_{i}=1\} \end{array}$$ (4)

$$\begin{array}{c} M=\{(s_{1},\cdots ,s_{t},\cdots ,s_{T}):s_{t}\in S,1\leq t\leq T\} \end{array}$$ (5)

### 3.2 Gumbel-softmax sampling

Since the communication channel involves discrete data, propagating errors from the Listener to the Speaker is not trivial. Addressing this issue requires either employing reinforcement learning techniques or utilizing continuous relaxation for the discrete communication channel. In our specific case, we use the Gumbel-Softmax trick, which [47] ensures that gradients can flow through the discrete communication channel. This technique has been employed in prior work, particularly in settings where the environmental dynamics need to be differentiable, as seen in [23].Given parameters *p* of a categorical distribution with dimensionality *K*, the sampling of differentiable one-hot encoded symbol *S* with *K* dimensions is described by Eq 6.

$$\begin{array}{c} S{\left(\text{log}\left(p\right)\right)}_{k}=\frac{e^{(\text{log}\left(p_{k}\right)+\epsilon )/\tau }}{\sum_{j=0}^{k}e^{(\text{log}\left(p_{j}\right)+\epsilon )/\tau }} \end{array}$$ (6)

*ϵ* is i.i.d. sample from Gumbel(0, 1) distribution, where *ϵ* = −log(−log(*u*)), and u∼U[0,1]. *τ* is the softmax temperature parameter. This makes the whole model differentiable end-to-end and allows the training of agents through direct backpropagation of errors.

### 3.3 Topological similarity

**Fig 2:**
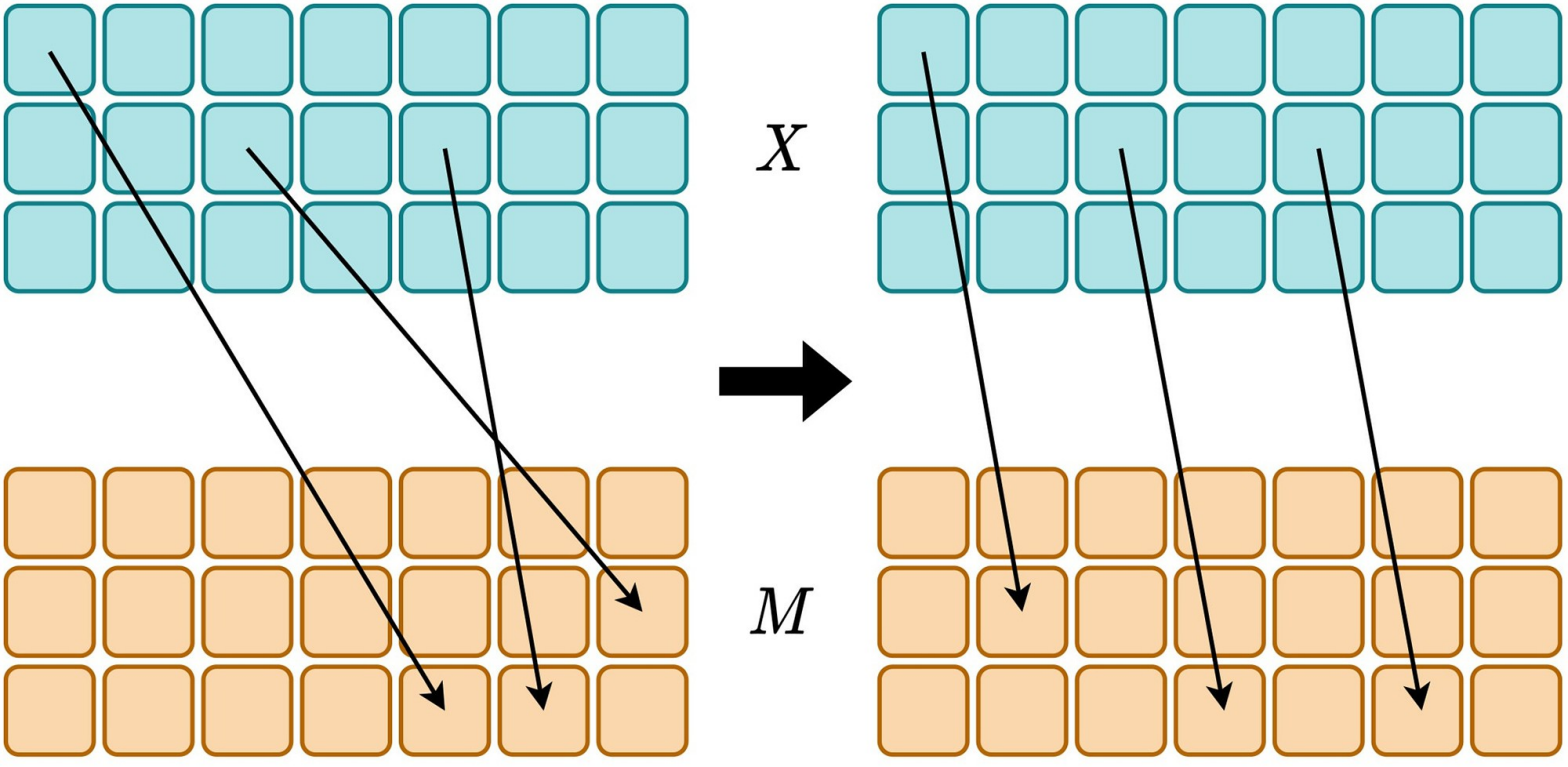
Intuition of topological similarity. The illustration on the left of the figure shows a hypothetical grounding map, which does not adhere to the notion of compositionality. The illustration on the right of the figure depicts what is expected when agents develop compositional languages. The top part of the image represents the inputs *X* while the bottom shows the messages *M*.

Given a set of inputs and the messages, the topological similarity is defined as the correlation between the pairwise input distances and the corresponding message distances [13, 15, 16]. Fig 2 depicts what we expect from incorporating topological similarity into agent training. The image on the left of Fig 2 depicts a non-compositional grounding map, where the distances between the messages do not mirror the distances between the inputs. Conversely, if agents are to adhere to a mapping scheme that respects compositionality, the result would resemble what is shown in the right of Fig 2. In this scenario, the distances between inputs would be closely mirrored in the corresponding messages, indicating an increase in topological similarity, which motivates us to optimize topological similarity for obtaining more structured languages.

To get the perfect estimation of topological similarity, we should calculate Pearson correlation using all possible ordered two-element subsets of both the inputs and corresponding messages (refer Table 1). However, we limit the scope to inputs and messages of a single mini-batch as calculating topological similarity for the entire dataset at once is not practically feasible for large datasets.

**Table 1:** Example input and message pairs.

Inputs	Messages	Input pairs	Message Pairs
*x*_1_, *x*_2_, *x*_3_	*m*_1_, *m*_2_, *m*_3_	(*x*_1_, *x*_2_), (*x*_1_, *x*_3_), (*x*_2_, *x*_3_)	(*m*_1_, *m*_2_), (*m*_1_, *m*_3_), (*m*_2_, *m*_3_)
*x*_1_, *x*_2_, *x*_3_, *x*_4_	*m*_1_, *m*_2_, *m*_3_, *m*_4_	(*x*_1_, *x*_2_), (*x*_1_, *x*_3_), (*x*_1_, *x*_4_), (*x*_2_, *x*_3_), (*x*_2_, *x*_4_), (*x*_3_, *x*_4_)	(*m*_1_, *m*_2_), (*m*_1_, *m*_3_), (*m*_1_, *m*_4_), (*m*_2_, *m*_3_), (*m*_2_, *m*_4_), (*m*_3_, *m*_4_)

We find ordered all possible two-element subsets for a given batch of inputs and Speaker messages. Hence, we get *n*(*n* − 1)/2 pairs for both the inputs and the messages.

**Fig 3:**
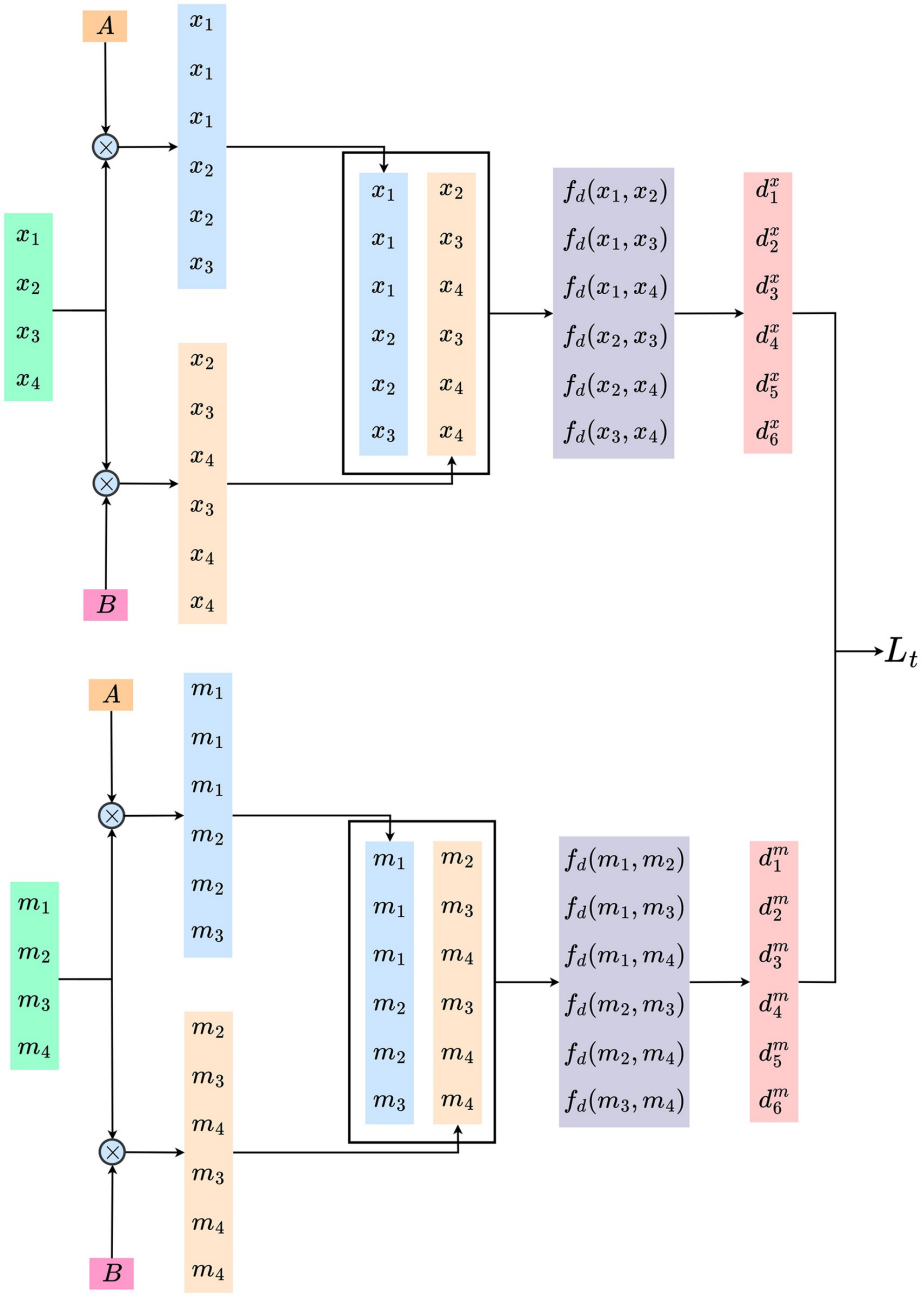
Estimating topological similarity. Example for estimating topological similarity for a batch size of four. We use matrices *A* and *B* to obtain all two-element subsets of the inputs and messages. The matrices are fixed for a given batch size. The entire process is differentiable.

Fig 3 shows the procedure for estimating topological similarity for a batch of four inputs and corresponding messages. As shown in the upper section of Fig 3, a mini-batch containing four input instances is multiplied by two matrices *A* and *B*, yielding two vectors, each with six elements. By combining these vectors, we generate all six possible two-element subsets for the mini-batch of inputs. Then, each of these pairs undergoes a distance calculation using the function *f*_*d*_, which leads to the generation of a distance vector with six elements corresponding to the batch of inputs. An identical procedure is conducted for the messages, resulting in a distance vector for the messages. These two distance vectors are then used to approximate the topological similarity $L_{t}$ between the inputs and the messages generated by the Sender. We use cosine similarity for implementing the function *f*_*d*_. The pairing process ensures that we obtain the maximum number of possible pairings, making the approximation of topological similarity more accurate and stable.

We now provide a more formal explanation of the procedure. Given a batch of inputs *X* = {*x*_1_, …, *x*_*n*_} and their related messages *M* = {*m*_1_, …, *m*_*n*_}, we obtain *X*′, *X*″ and *M*′, *M*″, by multiplying the inputs and messages with matrices *A* and *B* as in Eq 7. Elements of both matrices *A* and *B* are determined by the batch size *n*. *X*′, *M*′ contains the first elements while *X*″, *M*″ contains the second elements of all the pairs. Table 1 showcases an example where pairs are generated for an input batch of size 4. Hence for this specific example in Table 1, *X*′ = (*x*_1_, *x*_1_, *x*_1_, *x*_2_, *x*_2_, *x*_3_) and *X*″ = (*x*_2_, *x*_3_, *x*_4_, *x*_3_, *x*_4_, *x*_4_). We calculate *M*′ and *M*″ similarly.

$$\begin{array}{c} X^{\prime }=A⊗X\;\;\;\;\;X^{\prime \prime }=B⊗X\;\;\;\;\;M^{\prime }=A⊗M\;\;\;\;\;M^{\prime \prime }=B⊗M \end{array}$$ (7)

We define pairing matrices *A* and *B* by Eqs 8, 9 and 10. Each matrix has a size of (*n*)(*n* − 1)/2 × *n*, since each matrix should produce (*n*/2)(*n* − 1) pairs.

$$\begin{array}{c} A={\left(a_{ij}\right)}_{1⩽i⩽(n)(n-1)/2,1⩽j⩽n}\;\;\;\;B={\left(a_{ij}\right)}_{1⩽i⩽(n)(n-1)/2,1⩽j⩽n} \end{array}$$ (8)

Next obtain the sequence *Z*^*A*^ = (*n* − 1), (*n* − 1) + (*n* − 2), …, (*n* − 1) + (*n* − 2) + … + 1, which is used to determine the elements of matrix *A* as in the Eq 9

$$\begin{array}{c} a_{ij}=\{\begin{array}{cc} 1 & \text{if}\;j=\text{min}\{k;z_{k}^{A}⩾i\}\\ 0 & . \end{array} \end{array}$$ (9)

We define a sequence *Z*_*m*_ = *m* + 1, …, *n* and concatenate such sequences to form *Z*^*B*^ = *Z*_1_, *Z*_2_, …, *Z*_*n*−1_. If individual integer elements of *Z*^*B*^ are indexed by *i*, then pairing matrix *B* is define as follows (refer Eq 10),

$$\begin{array}{c} b_{ij}=\{\begin{array}{cc} 1 & \text{if}\;j=z_{i}^{B}\\ 0 & . \end{array} \end{array}$$ (10)

We create our pairing matrices once and store them in the memory. Both matrices are detached from the computational graph and remain constant throughout the training. We depict the implementation of the distance metric *f*_*d*_ by Eqs 11 and 12 and Algorithm 1. As the first step, we calculate the similarity between pairs as in Eq 11 and Algorithm 1, which is then used to obtain the distance vectors as in Eq 12.

$$\begin{array}{c} S^{X}=\text{Sim}\left(X^{\prime },X^{\prime \prime },V,A\right)\;\;\;\;\;S^{M}=\text{Sim}\left(M^{\prime },M^{\prime \prime },S,T\right) \end{array}$$ (11)

**Algorithm 1** Similarity Algorithm :- Sim(*P*, *Q*, *l*_1_, *l*_2_)

1: $A\leftarrow {\text{Reshape}}_{\frac{n}{2}\left(n-1\right)l_{2}\times l_{1}}\left(P\right)$    ⊳$\text{dim}\left(P\right)=\text{dim}\left(Q\right)=\frac{n}{2}\left(n-1\right)\times l_{1}l_{2}$

2: $B\leftarrow {\text{Reshape}}_{\frac{n}{2}\left(n-1\right)l_{2}\times l_{1}}\left(Q\right)$

3: *S* ← CosineSimilarity(*P*, *Q*)

4: $S\leftarrow {\text{Reshape}}_{\frac{n}{2}\left(n-1\right)\times l_{2}}\left(S\right)$

5: **return** *S*

This leads to two distance vectors *D*^*X*^ and *D*^*M*^

$$\begin{array}{c} D^{X}\left(i\right)=1-\frac{1}{A}\sum_{j=1}^{A}s_{i,j}^{X}\;\;\;\;\;D^{M}\left(i\right)=1-\frac{1}{T}\sum_{j=1}^{T}s_{i,j}^{M} \end{array}$$ (12)

Finally, we represent the topological similarity as the Pearson correlation between distance vectors *D*^*X*^ and *D*^*M*^ as in Eq 13.

$$\begin{array}{c} L_{t}=1-\frac{\frac{n}{2}\left(n-1\right)\sum_{}^{}d_{i}^{x}d_{i}^{m}-\sum_{}^{}d_{i}^{x}\sum_{}^{}d_{i}^{m}}{\sqrt{\frac{n}{2}\left(n-1\right)\sum {\left(d_{i}^{x}\right)}^{2}-{\left(\sum d_{i}^{x}\right)}^{2}}\sqrt{\frac{n}{2}\left(n-1\right)\sum {\left(d_{i}^{m}\right)}^{2}-{\left(\sum d_{i}^{m}\right)}^{2}}} \end{array}$$ (13)

Spearman correlation, which was used to measure the correlation in earlier work [15], is not directly differentiable due to the involvement of ranking operation. Hence, we use Pearson correlation in our approach. Soft ranking methods, which are differentiable, exist, but we do not investigate such approaches for the sake of simplicity.

When training with topological similarity loss, we apply the reconstruction loss alone for a selected number of initial batches. We apply the topological similarity loss after exempting this initial set of batches. Initial exemption stabilizes the performance across random seeds. In our earlier experiments, where the exemption was not implemented, we observed a significant variation in test performance. The process of exempting causes the models to be updated towards a specific point, which favors the reconstruction loss. Therefore, the priority is placed on the reconstruction loss. We apply exempting in all of our trials as required, as we have empirically determined that it is effective for various channel configurations. Due to the success of the exempting strategy, we do not investigate other methods, such as weighing the losses using appropriate loss coefficients.

### 3.4 Sparse grounding map

**Fig 4:**
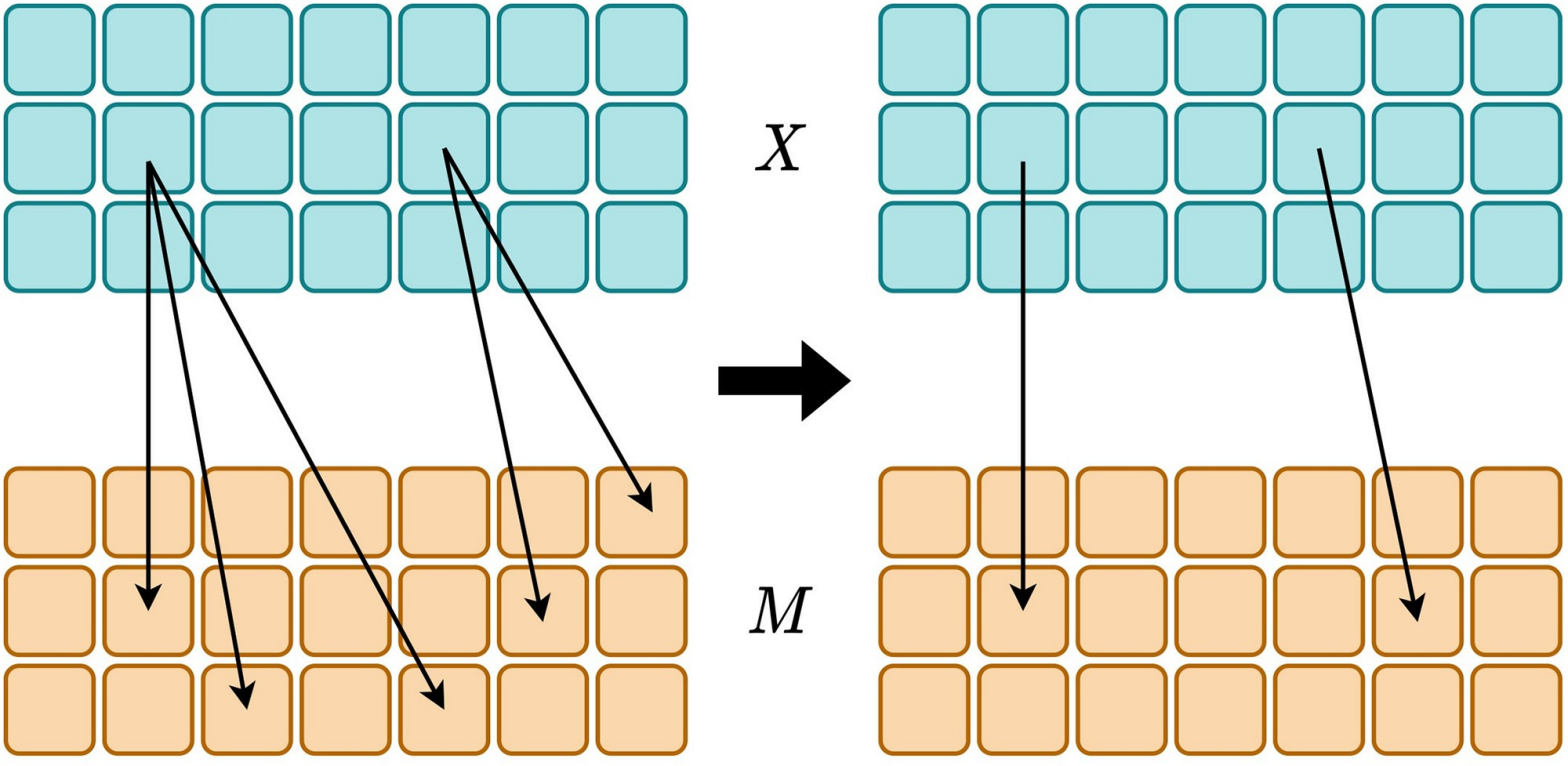
Intuition of sparse groundings. A sparse grounding map contains strong but few connections between inputs and messages. In comparison, such mappings are simpler and exhibit less entanglement. The illustration on the left side contains surplus connections, an occurrence that we intend to remove. The inputs *X* are represented at the top of the image, while messages *M* are indicated at the bottom.

Most of the language emergent systems do not follow the principle of parsimony. The principle of parsimony implies that a smaller set of solid connections should be favored over a complex set of connections, given that task completion is not negatively affected. Sparsifying the grounding map prunes surplus links between inputs and messages. A grounding map becomes increasingly complex if it develops multiple connections arbitrarily during the learning process. We demonstrate our intuition behind the sparse grounding map in Fig 4. The illustration on the left has multiple redundant connections from inputs to messages. We intend to remove such surplus connections and simplify the mappings similar to what is illustrated on the right of the figure.

**Fig 5:**
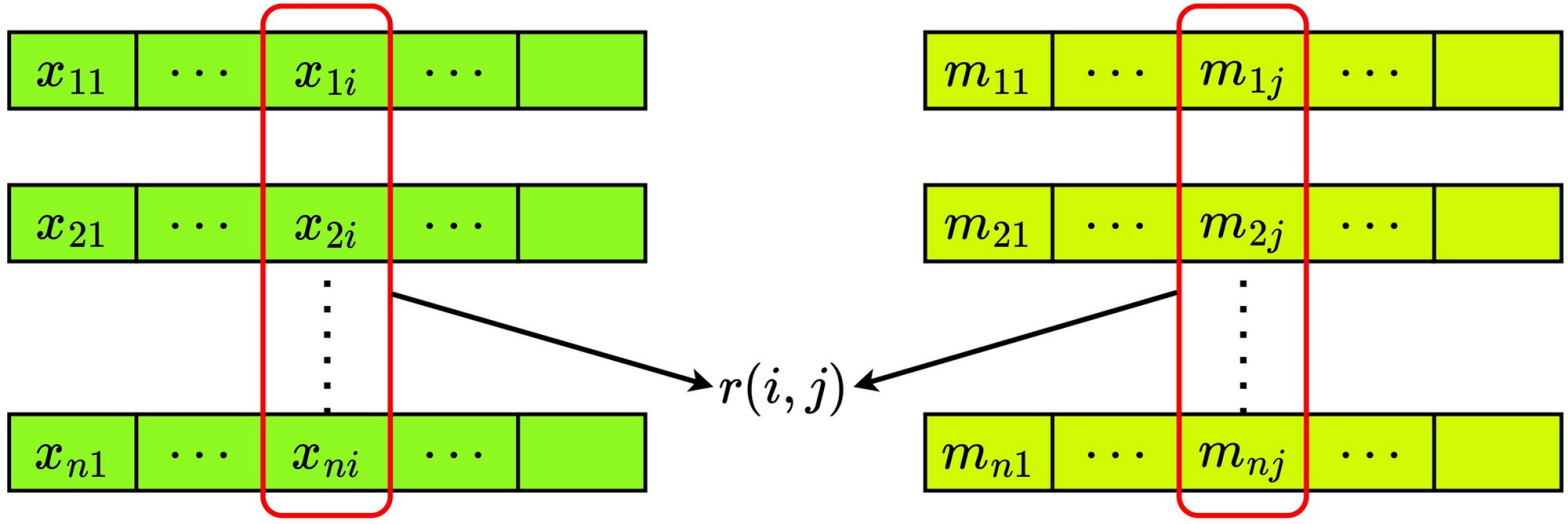
Calculating sparse grounding. We calculate correlation across the entire input and message vectors considering each index, which gives an approximation for the complete grounding map.

The optimal metric for evaluating the link strength between concepts and symbols is mutual information. Nevertheless, computing mutual information in a differentiable way is not trivial. Complex differentiable approximations of mutual information exist. However, for simplicity, we use Pearson correlation to approximate the linkage between concepts and symbols. Data in our experiments consists of multiple attributes and a fixed number of values for each attribute. Moreover, messages are formed by repeatedly concatenating symbols sampled from a fixed-size vocabulary. Consequently, we compute correlations between each value in every attribute and each symbol at every position of the Speaker’s messages (see Fig 5 and Eq 14). We use a single mini-batch for estimating the sparsity, similar to the calculation of topological similarity.

$$\begin{array}{c} r\left(i,j\right)=\frac{n\sum_{}^{k}x_{ki}m_{kj}-\sum_{}^{k}x_{ki}\sum_{}^{k}m_{kj}}{\sqrt{n\sum_{}^{k}{\left(x_{ki}\right)}^{2}-{\left(\sum_{}^{k}x_{ki}\right)}^{2}}\sqrt{n\sum_{}^{k}{\left(m_{kj}\right)}^{2}-{\left(\sum_{}^{k}m_{kj}\right)}^{2}}} \end{array}$$ (14)

As defined in Eq 15, we obtain the *L*1 loss for the correlations in the entire grounding map.

$$\begin{array}{c} L_{s}=\sum^{i}\sum^{j}|r(i,j)| \end{array}$$ (15)

In our experiments, we found that applying this loss alone may cause all links in a grounding map to vanish, especially under high learning rates or large sparse loss coefficients *C*_*s*_. As a solution, we add another term to the above loss function (refer Eq 16), which protects stronger correlations from decaying. We use the LogSumExp (LSE) trick to approximate a smooth maximum.

$$\begin{array}{c} L_{s}=\frac{1}{VA}(\sum^{i}\sum^{j}|r(i,j)|-\frac{1}{\tau }\sum^{i}\;\text{log}\;(\sum^{j}e^{|r(i,j)|\tau })) \end{array}$$ (16)

### 3.5 Experimental setup

We carry out an extensive set of experiments to investigate the effect of functional pressures. Although modeled as an auxiliary loss and employed as a functional pressure, our notion of sparsifying groundings is a novel concept, which requires thorough experimenting to establish advantages. Since it has not been previously studied, we opt to do multiple experiments to establish its connection to generalization and learning speed and how it is affected by the training data availability, batch size, and learning rate. Topological similarity and compositionality have been discussed in prior literature. Nonetheless, as we model it as a differentiable auxiliary loss, thorough experimenting is again needed to determine its utility.

We conduct preliminary experiments to determine the learning rates and weight decay. Based on the results, learning rate and weight decay are constrained within [1 *e* − 3, 5 *e* − 3, 1 *e* − 2] and [1 *e* − 8, 1 *e* − 6, 1 *e* − 4], respectively. Unless otherwise stated, our common experimental protocol for any given occasion is as follows. We use 80% of data for training and keep the remaining 20% for testing. We repeat each and every experiment for three random seeds (1, 2, 3), train our agents for 100 epochs, and use a learning rate of 0.01 and a batch size of 128. Agents undergo four methods of training for any given occasion. First, agents are trained without both the functional pressures and weight decay for measuring the baseline performance. Secondly, we train agents with multiple weight decay values and record the best performance so the performance of the functional pressures can be compared with a commonly available regularization technique. Finally, without using weight decay, agents are trained with each one of the functional pressures separately. We outline the abovementioned methods in Table 2.

**Table 2:** Explanation of agent training methods.

Method	Explanation	Primary objective loss	Functional pressure	Weight decay
Baseline	Baseline	Reconstruction loss	None	None
Weight decay	Baseline + Weight decay	Reconstruction loss	None	Yes
Topsim	Baseline + Topological similarity	Reconstruction loss	Topological similarity loss	None
Sparse	Baseline + Sparse correlation	Reconstruction loss	Sparse grounding loss	None

We train our agents using four different methods, each having one unique difference compared to the Baseline.

For each method, we record the highest test accuracy and the epoch in which it is reached and then obtain the average of those across the three random seeds. We set *C*_*s*_ = 0.8 and *C*_*t*_ = 0.5. When training with topological similarity, the initial 1–20 batches are only trained with the reconstruction loss $L(x,x^{\prime })$. After exempting these first batches, agents train to minimize both the reconstruction and the topological similarity losses. For all methods, we stop training when agents reach 99.0% test accuracy.

Our hypothesis revolves around the notion that the development of a coherent structure during the emergence of language should manifest as heightened test accuracy. Furthermore, the primary utility of emergent languages lies in achieving the intended objective, making gains in test accuracy a paramount objective. When agents construct simple and coherent languages, we anticipate observing increased convergence rates. In addition, the inherent structure and simplicity of these languages should facilitate better generalization, especially when training data is limited. We seek to explore the performance of our algorithms under varying experimental conditions, such as different learning rates and batch sizes. If the advantages remain consistent across diverse batch sizes and learning rates, it would suggest that our approaches are effectively providing regularization and enhancing generalization. To validate this hypothesis, we devise a series of experiments, briefly outlined below.

#### 3.5.1 Experiment set 1

We do our first set of experiments with the *X*(10, 5) input dataset containing 10^5^ unique samples and use three channel configurations ranging from *M*(10, 6), *M*(10, 8) to *M*(100, 8).

#### 3.5.2 Experiment set 2

We compare the performance of our proposed functional pressures with that of the existing work [4]. We do not conduct baseline or weight decay experiments as we intend to compare the performance of our functional pressures with that of the existing work. We train our agents for 600 epochs and use 90% percent of data for training the agents, similar to [4]. All other hyperparameters and aggregation of results are similar to what is explained in Section 3.5. For the prior work, [4], agents train for 3000 epochs.

#### 3.5.3 Experiment set 3

We then run experiments with *X*(10, 5) input dataset with different proportions of training data. We select 90% of data for testing while only keeping 10% for training. We train agents for 100 epochs and use the channel configurations *M*(10, 6), *M*(10, 8) and *M*(100, 8). Other hyperparameters are not changed.

#### 3.5.4 Experiment set 4

We train our agents using three different batch sizes and learning rates to investigate whether the advantage offered by our functional pressures is stable. For a further tight evaluation, we keep hyperparameters relevant to functional losses constant throughout the entire experiment regardless of the batch size, learning rate, and channel configuration. We still search over multiple weight decay values as in the common protocol. Not tuning loss coefficients *C*_*t*_ and *C*_*s*_ may yield sub-optimal results for functional pressures under some configurations. However, our results show that functional pressures perform above the baselines and weight decay even under such sub-optimal hyperparameter selection.

#### 3.5.5 Experiment set 5

We compare the performance of our algorithms in an out-of-domain generalization task proposed in [48]. We do not conduct experiments relating to weight decay and baseline. We use *C*_*t*_ = 0.5 and *C*_*s*_ = 0.2, and all other hyperparameters are not changed.

## 4. Results

### 4.1 Experiment set 1

**Fig 6:**
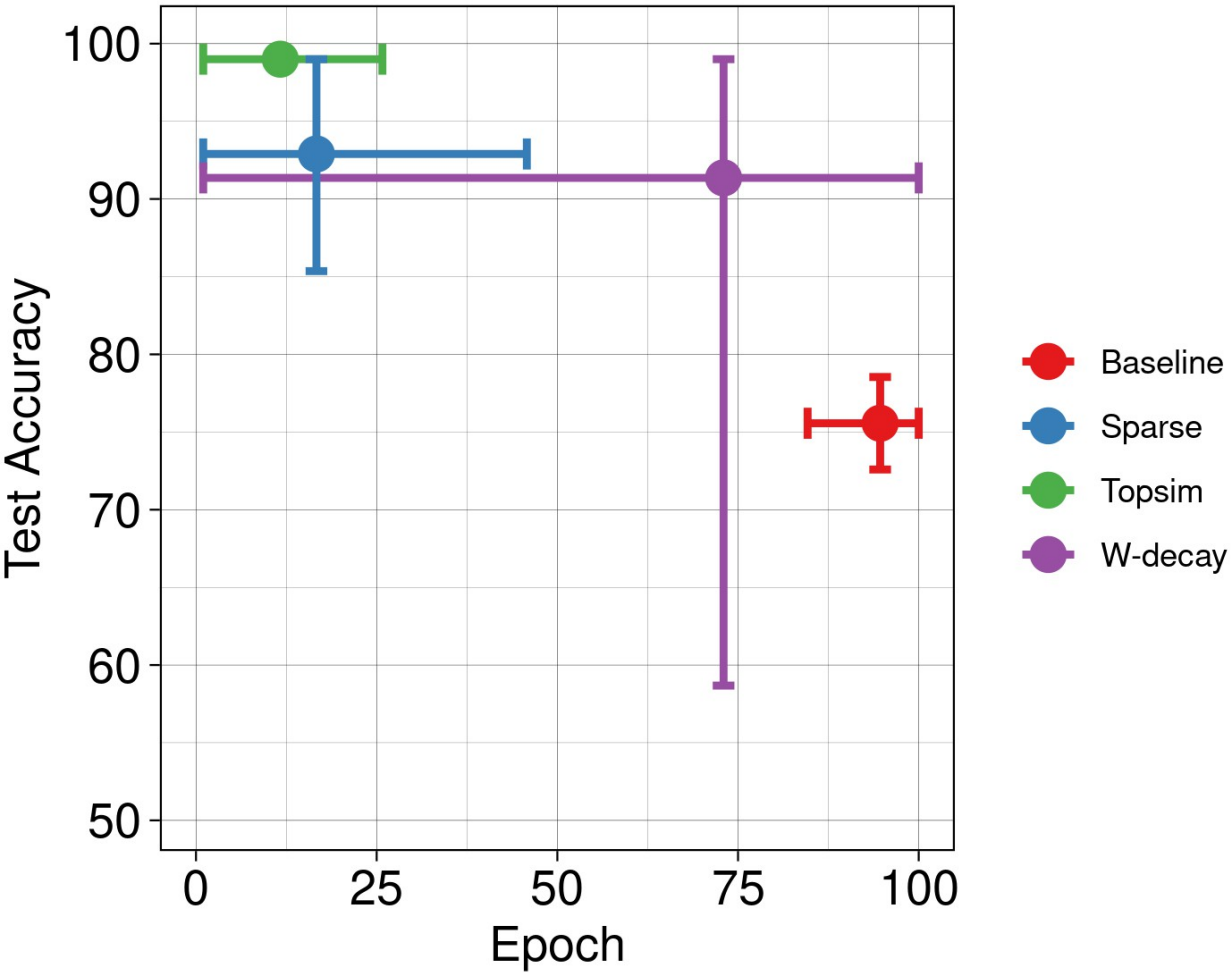
Test accuracy Vs epochs at M(10, 6). Our approaches perform well under limited channel capacities, providing better test accuracy and convergence speed.

**Fig 7:**
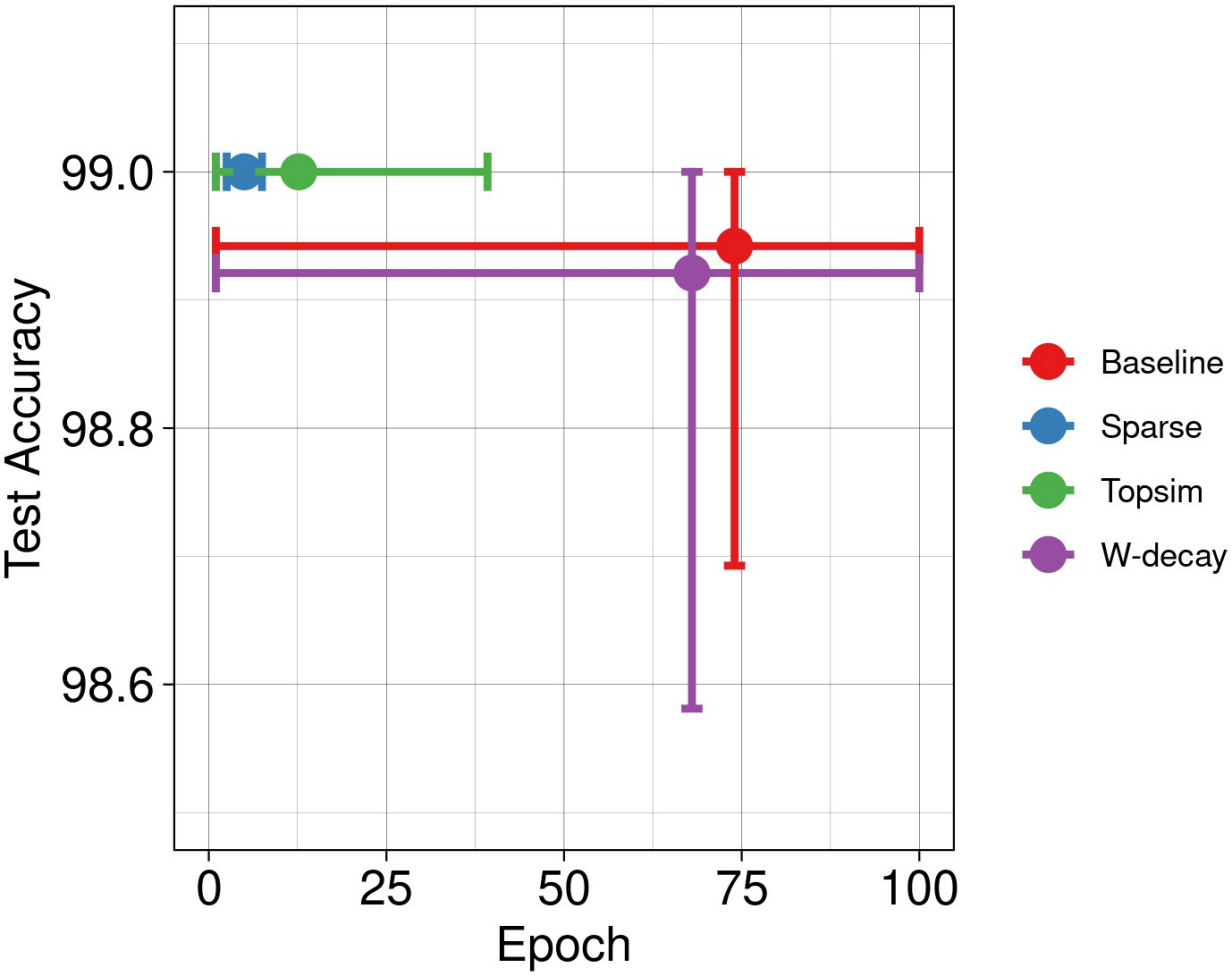
Test accuracy Vs epochs at M(10, 8). The comparative improvement in test accuracy attributed to functional pressures becomes relatively modest as channel capacity increases. However, our approaches exhibit significantly earlier convergence.

In conjunction with other methods, the results of our functional pressures are depicted in Fig 6. Results show enhancements in test accuracy and learning speed compared to the other two approaches. Next, we increase the message length to 8 symbols while maintaining the vocabulary size. Fig 7 shows significant improvements in test accuracy across all methods. While the baseline and weight decay approaches achieve near-perfect test accuracy, our methods still lead to a more rapid convergence.

**Fig 8:**
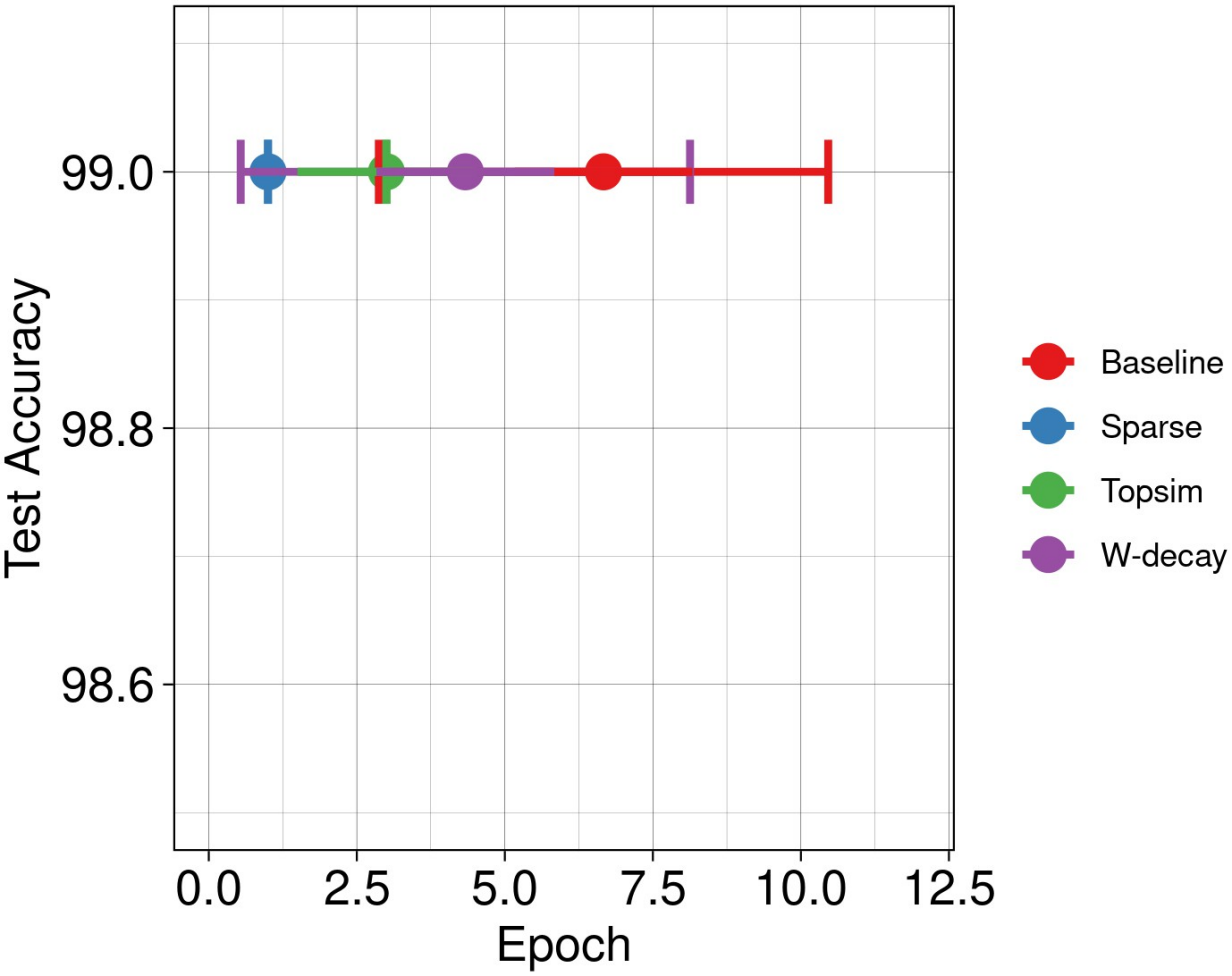
Test accuracy Vs epochs at M(100, 8). While there is no substantial disparity in test accuracy, models trained with our approaches exhibit an accelerated convergence rate.

Then, we conduct experiments using a channel with a much larger capacity M(100, 8), which expands the available vocabulary size by 10 and the capacity by 10^8^. Such larger channel capacities offer agents greater flexibility in message construction, posing a less constrained discrete bottleneck. Hence, all models achieve perfect test accuracy, with minimal variations (Fig 8). Notably, all approaches exhibit improved convergence speeds compared to the smaller channel capacities.

### 4.2 Experiment set 2

Table 3 compares test accuracy between our approach and a previous study [4] that utilized the same datasets and channel configurations. In the prior study, agents were trained for 3000 epochs, while we only train our agents for 600. Within our experiments, we incorporated two scenarios characterized by tightly constrained channel capacities, namely *M*(5, 3) and *M*(10, 2), where the size of the value-attribute dataset equals the channel capacity. In these configurations, all methods show a considerable decline in test accuracy. Nevertheless, agents trained with auxiliary losses provide the best results. Importantly, we do not tune the coefficients *C*_*t*_ and *C*_*s*_. Hence, there may be room for further improvements by fine-tuning these coefficients to better align with the more restricted channel configurations. Additionally, it is worth noting that the number of individual data points in *X*(4, 4) is quite limited, totaling only 256, causing a learning bottleneck for agents. Under all configurations functional pressures provide the best results.

**Table 3:** Test accuracy comparison with prior work.

Input	Capacity	Test Accuracy (Chaabouni el al.)	Test Accuracy (Ours, Topsim)	Test Accuracy (Ours, Sparse)
X(4,4)	*M*(5, 4)	37.5±0.0	**77.6 ± 22.1**	**86.4 ± 20.8**
X(5,3)	*M*(5, 3)	16.6±0.0	**24.2 ± 7.5**	**54.4 ± 34.9**
X(10,2)	*M*(10, 2)	20.8±7.2	**40.6 ± 13.2**	**52.3 ± 3.3**
X(50,2)	*M*(50, 3)	83.5±4.0	**98.1 ± 1.5**	**98.2 ± 0.9**
X(50,2)	*M*(100, 2)	76.1±21.4	**96.1 ± 3.7**	**96.2 ± 0.0**

We perform tests across multiple input and channel configurations and compare those results with prior work, which used identical datasets and channel configurations.

### 4.3 Experiment set 3

**Fig 9:**
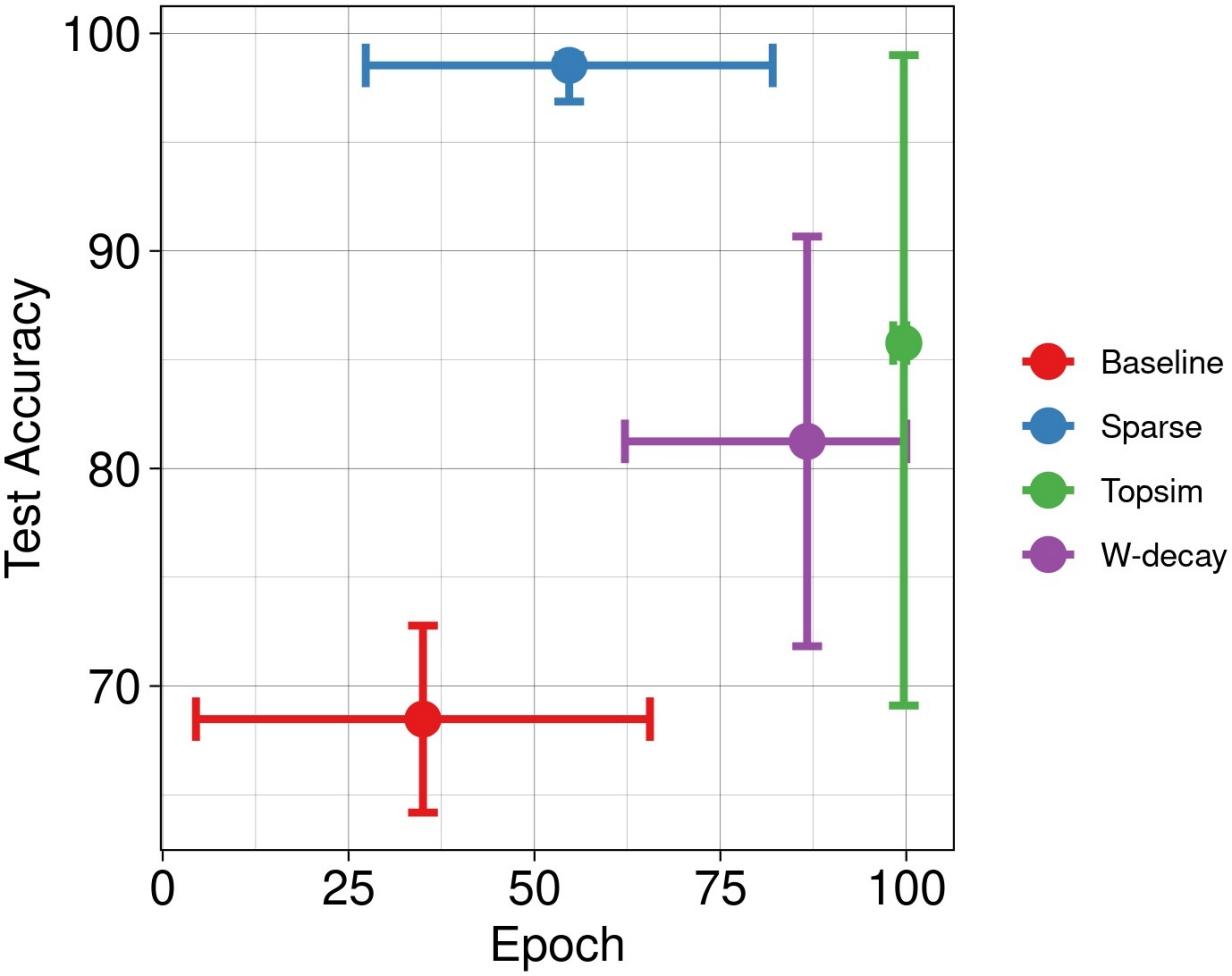
Test accuracy Vs epochs for reduced training data at M(10, 6). Our methods surpass results obtained with weight decay and baselines.

**Fig 10:**
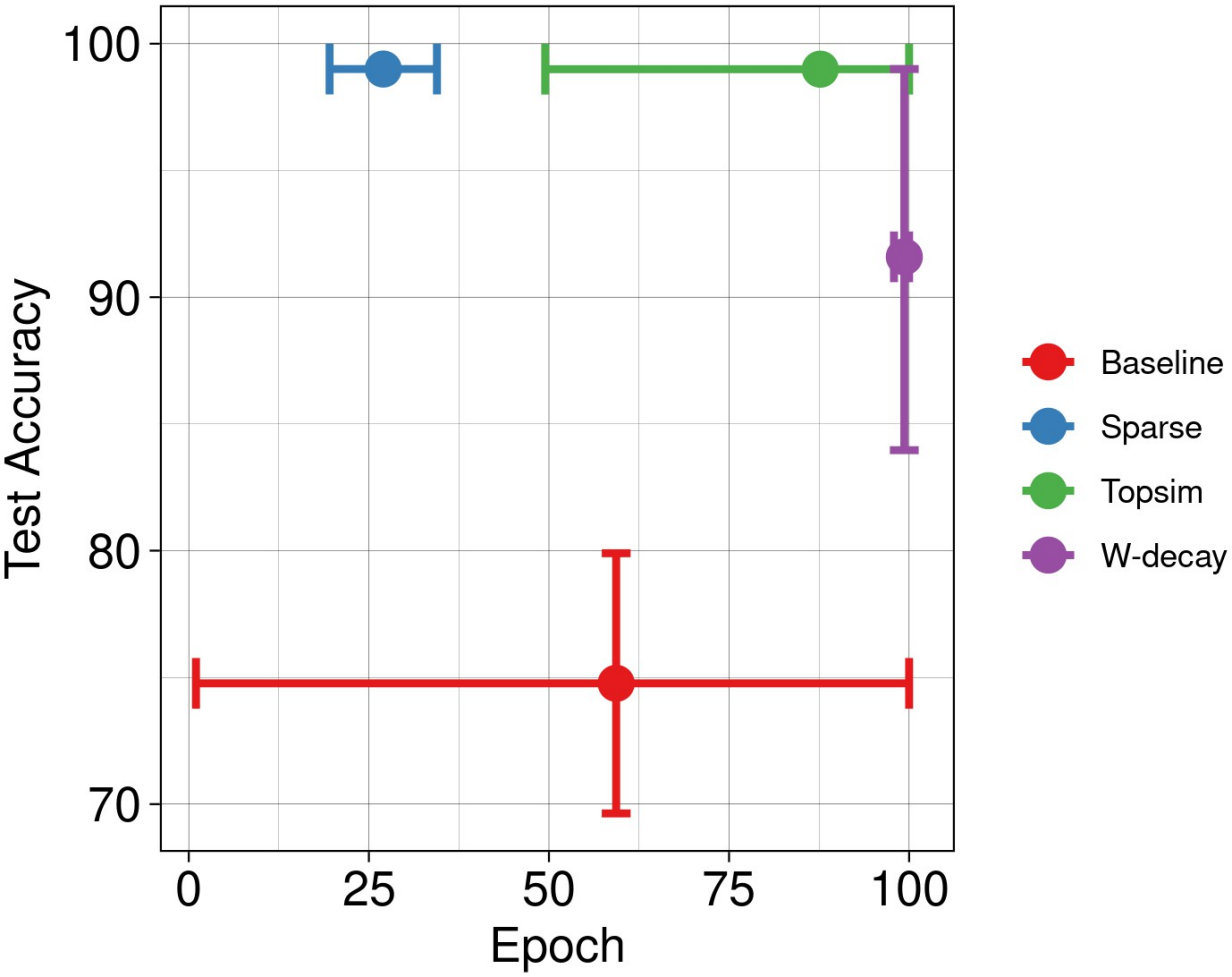
Test accuracy Vs epochs for reduced training data at M(10, 8).

We conduct a new set of experiments with identical input datasets and channel configurations to the Experiment set 1 but with a much lower percentage of training data. Specifically, we reserve 90% of the data for testing purposes and use only 10% for training. Figs 9 and 10 illustrate a decrease in test accuracy for both baseline experiments and weight decay compared to the previous scenarios. In this case, functional pressures provide better test accuracy than other methods.

**Fig 11:**
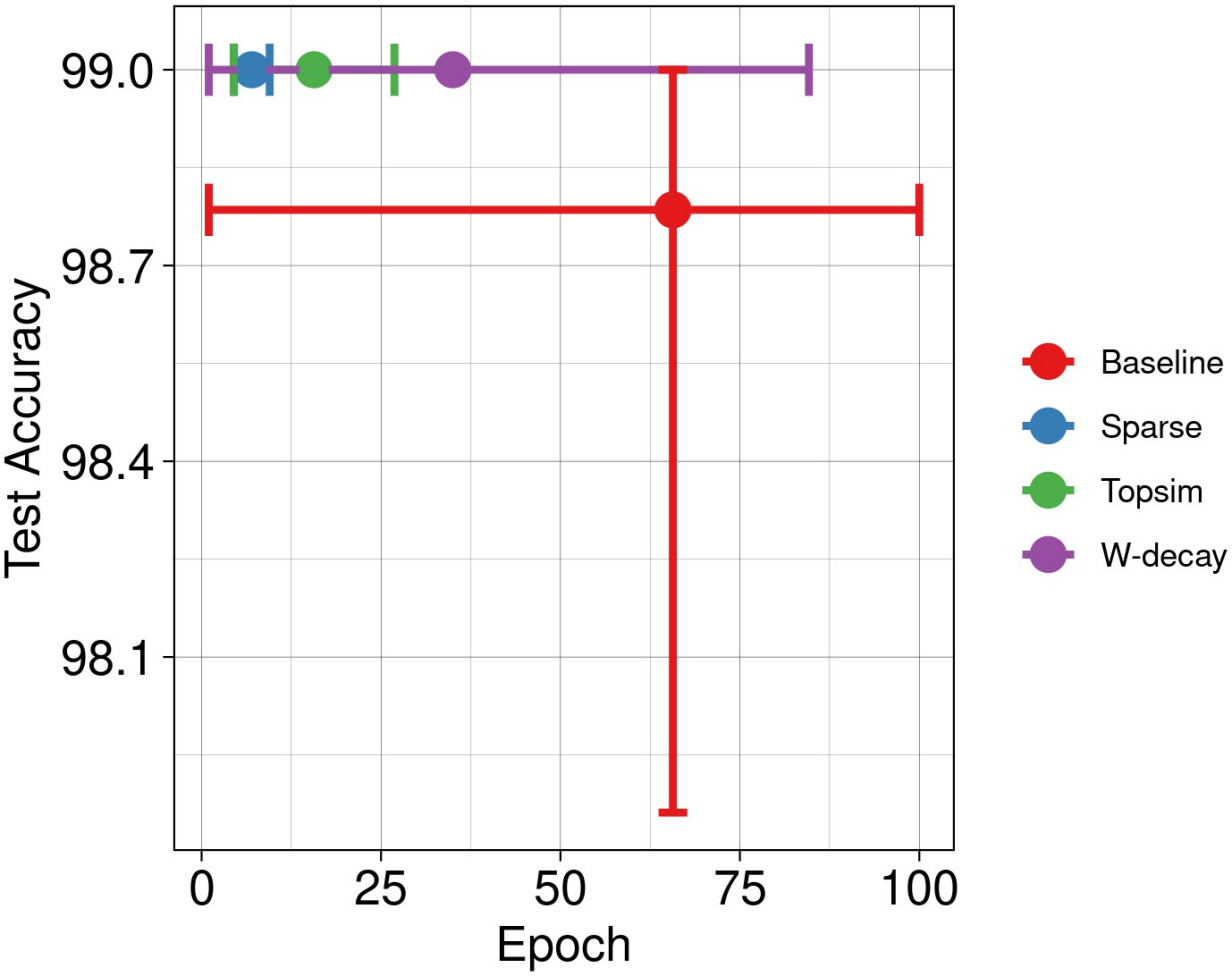
Test accuracy Vs epochs for reduced training data at M(100, 8). Overwhelmingly large channel capacity can mitigate the negative effects of low training data. Although models do not differ much in test accuracy, learning speed is higher for the models trained with functional pressures.

Nonetheless, the substantial freedom conferred by excessive channel capacity can effectively mitigate the decline in test accuracy stemming from limited training data, as illustrated in Fig 11. These results emphasize that the ratio of channel capacity to input data size is pivotal in determining model performance, regardless of other factors. When channel capacity exceeds the dimensions of the input dataset, even baseline models tend to exhibit commendable generalization. Notably, functional pressures still exhibit faster learning rates in such situations than other approaches, often requiring approximately 50% fewer epochs to achieve convergence.

### 4.4 Experiment set 4

We investigate the stability of employing functional pressures by varying batch size, learning rate, and input and channel configurations. Table 4 shows all the experimental configurations used in this section.

**Table 4:** Experiment settings.

X(V, A)/M(S, T)	64			128			256		
	1e − 2	5e − 2	1e − 3	1e − 2	5e − 2	1e − 3	1e − 2	5e − 2	1e − 3
X(100,2)/M(100,3)	x	x	x	x	x	x	-	-	-
X(10,4)/M(10,6)	x	x	x	x	x	x	x	x	x
X(34,3)/M(41,5)	x	x	x	x	x	x	x	x	x

We perform tests using settings indicated by ‘x’ for Section 4.4. We omit settings indicated by ‘-’ due to resource constraints.

Table 5 displays an overview of test accuracy and convergence across different batch sizes. The presented results are an average across various learning rates and input/channel configurations, except *X*(100, 2). The exclusion of *X*(100, 2) ensures comparability across all batch sizes, as outlined in Table 4. Our functional pressures rely on the correlation approximation within a single batch. Therefore, as batch size decreases, samples available for correlation calculation also decrease. Nevertheless, the results indicate that both pressures are resilient against varying batch sizes, where functional pressures consistently yield superior performance in test accuracy and convergence speed.

**Table 5:** Average results across varying batch size.

Size	64		128		256	
	Accuracy	Epoch	Accuracy	Epoch	Accuracy	Epoch
Baseline	92.9 ± 4.1	76.1 ± 13.2	94.7 ± 2.8	82.5 ± 12.0	95.2 ± 2.5	73.8 ± 14.6
W-decay	97.7 ± 1.0	79.4 ± 13.8	97.4 ± 1.1	78.3 ± 13.2	97.2 ± 1.0	80.1 ± 11.9
Sparse	99.0 ± 0.2	32.2 ± 13.9	99.0 ± 0.1	26.0 ± 10.3	99.0 ± 0.2	30.7 ± 10.4
Topsim	98.7 ± 0.9	59.2 ± 9.9	98.9 ± 0.6	50.3 ± 10.9	99.1 ± 0.1	51.2 ± 10.2

We do not average over the configuration *X*(100, 2), *M*(100, 3) to make the results comparable.

Results on varying learning rates are shown in Table 6. Notably, we do not detect any noticeable adverse effects on the performance of our methods. Among these methods, training with sparse grounding pressure consistently outperforms others across all configurations, closely followed by training with topological similarity. We observe that both functional pressures provide consistent increases in convergence speed regardless of the experimental configuration. Employing sparse grounding loss provides the best speed, and it mostly outperforms all other methods.

**Table 6:** Average results across varying learning rates.

Learning Rate	1e − 3		5e − 3		1e − 2	
	Accuracy	Epoch	Accuracy	Epoch	Accuracy	Epoch
Baseline	89.0 ± 6.1	89.9 ± 5.5	90.7 ± 3.8	66.5 ± 11.8	88.3 ± 3.8	69.9 ± 12.2
W-decay	89.1 ± 6.3	93.9 ± 2.2	92.8 ± 3.4	66.5 ± 11.2	94.7 ± 2.7	74.8 ± 12.9
Sparse	96.5 ± 2.3	51.9 ± 12.9	98.4 ± 0.6	37.1 ± 13.3	98.3 ± 0.6	45.6 ± 16.2
Topsim	91.0 ± 6.1	63.5 ± 9.3	96.5 ± 2.2	56.0 ± 11.3	97.0 ± 1.4	66.5 ± 10.9

### 4.5 Experiment set 5

We conduct another experimental setup to validate the out-of-domain generalization of our methods. Our experimental configuration in this scenario closely resembles [48]. Specifically, we choose the value-attribute environment *X*(100, 2) and a channel configuration of *M*(100, 3). The out-of-domain test set encompasses all instances where the value 0 is present, excluding just three cases: (0, 0), (0, 1), and (1, 0). These three examples are incorporated into the training set, while the remaining data not included in the out-of-domain test set are also part of the training set.

Table 7 illustrates the accuracy of different approaches on the out-of-domain test set. Both of our methods exhibit superior performance compared to other techniques. In contrast to the [48], which employs an encoder-decoder architecture enriched with attention, our models stand out due to their simplicity. While we did not test encoder-decoder architectures or models incorporating attention mechanisms, we anticipate that using such models and fine-tuning coefficients could yield superior results compared to the current out-of-domain generalization results we show here.

**Table 7:** Test accuracy for out-of-domain test set.

Model	Train	In Domain Test	Out of Domain Test
Auersperger et al. (2022)	99.0 ± 3.0	98.0 ± 5.0	42.0 ± 27.0
Chaabouni et al. (2020)	99.0 ± 7.0	91.0 ± 9.0	0.0 ± 1.0
**Ours (Topsim)**	**100.0 ± 0.0**	**99.8 ± 0.7**	**80.6 ± 12.5**
**Ours (Sparse)**	**100.0 ± 0.0**	**99.9 ± 0.5**	**79.1 ± 9.5**

We test the out-of-domain generalization of our methods. Our agents show better test accuracy despite having a simpler architecture.

## 5. Analysis

In this section we explore qualitative and quantitative characteristics of the emerged languages. We expect distinctive linguistic properties to emerge when agents are trained with functional pressures. However, manually analysing the connection between inputs and messages is challenging. In Table 8, we show sample messages that agents produced for 10 test inputs, and it is not immediately evident how agents represent the input data in their messages. Therefore, we examine the developed languages using approaches that involve holistic visualizations and probabilistic context-free grammar.

**Table 8:** Agent generated messages under all four methods.

Inputs				Messages			
A1	A2	A3	A4	Baseline	Weight decay	Topsim	Sparse
2	0	0	2	HJFEIE	FBJDFG	IFIIJJ	FJEAGJ
7	6	3	2	IFIIHJ	JFFFJF	GHDDJJ	BBJBGH
9	7	4	9	CJIDAG	EHIIFJ	BDHHCC	BAIJBH
0	7	3	2	FIFJFE	EJEDEF	FDDDJJ	GAJBBH
3	1	6	6	GHGHFJ	BECIEI	EEFFEE	IECIGI
5	5	6	1	FFAFHI	IHCCJC	JAFFDD	CEEIGB
7	0	8	2	GJDEAE	BFGGAG	GFBBJJ	BBJGAJ
3	1	3	3	IHHGFD	BEFEDD	EEDDAA	IEJBCI
8	9	8	2	GDGJGA	AAIEAG	AIBBJJ	ECJGAH
7	1	0	4	HHGFIF	BFFFFF	GEIIFF	FBEAHI

Inputs are from a *X*(10, 4) dataset, while the messages correspond to a *M*(10, 6) channel. *A*1, *A*2, *A*3, and *A*4 denote the four attributes of the dataset, and each number represents the value taken for that particular attribute for that instance. We represent vocabulary with letters as there are only 10 distinct symbols. For larger vocabularies, mapping with letters may not be possible.

### 5.1 Visual analysis of language groundings

In this section, we do two types of visualizations to identify patterns within emerged languages. For this study, we select the value-attribute environment *X*(10, 4) and two channel configurations *M*(10, 6) and *M*(10, 8). We train agents using all four training methods and obtain all agent messages for the entire set of test inputs, accounting for 2000.

Then for any given attribute *a* where 1 ≤ *a* ≤ *A* and any given message position *t* where 1 ≤ *t* ≤ *T*, we calculate symmetrical uncertainty (SU) between *a* and *t*, SU(*a*, *t*) to function as a proxy of how strongly a given attribute is grounded or connected with a particular position in messages, leading to a *A* × *T* matrix consisting of symmetrical uncertainty values. Symmetrical uncertainty is a normalized measure with a range of [0, 1]. A value of 1 indicates that knowledge of one variable completely predicts the other, and a 0 indicates that two variables are independent [49]. We plot the SU map separately for all four methods and channel configurations.

Similarly, for any given value *v*_*a*_ where *v*_*a*_ ∈ {0, 1} at attribute *a* and any given symbol *s*_*t*_ where *s*_*t*_ ∈ {0, 1} at message position *t*, we calculate the SU(*v*_*a*_, *s*_*t*_) to measure grounding strength between individual values and symbols. *v*_*a*_ indicates the presence of value *v* at attribute *a* and *s*_*t*_ indicates the presence of symbol *s* at position *t* in the messages. The above calculations produce a matrix of size *VA* × *ST*.

#### 5.1.1 Representation of values and attributes in messages

**Fig 12:**
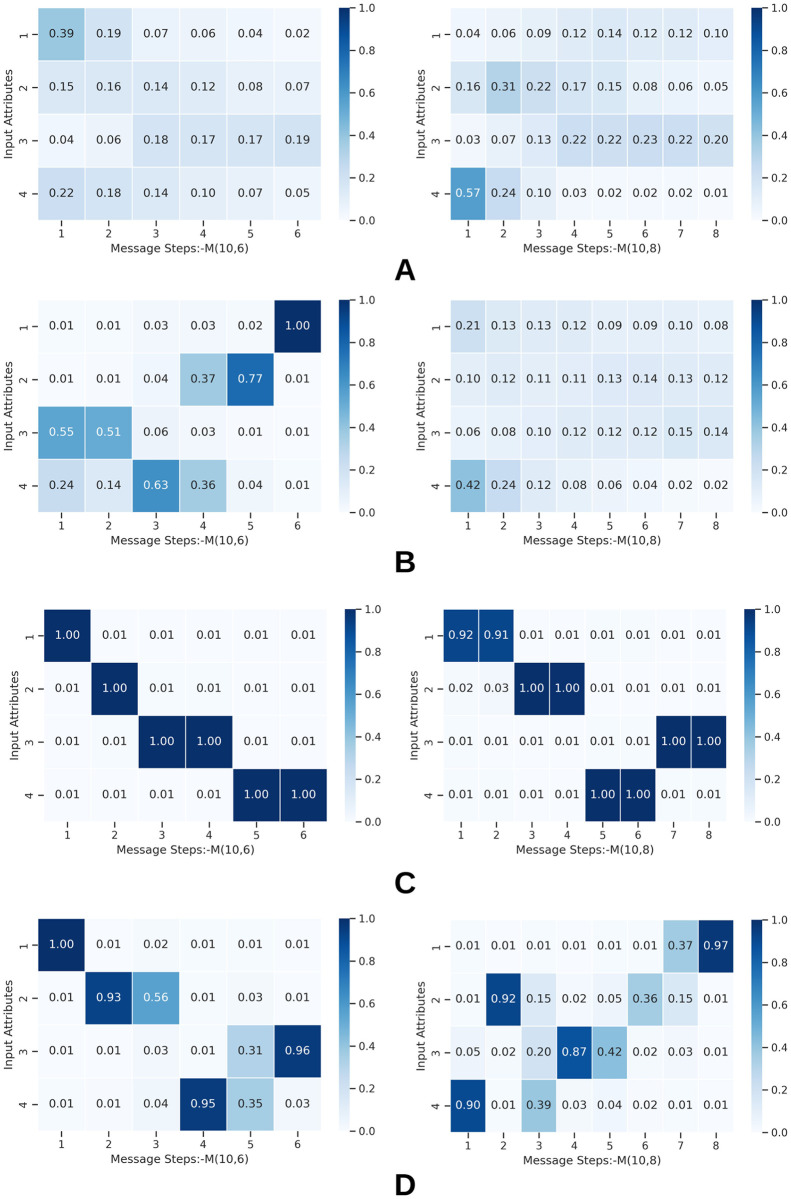
Message position to attribute linkage measured by symmetric uncertainity at various channel capacities. Each panel displays the linkage strength between attributes and message positions. Each row depicts a different agent training method. A: Baseline. B: W-decay. C: Topological similarity. D: Sparse grounding. The horizontal axis and vertical axis of each map represent message positions(steps) and input attributes, respectively.

Fig 12 represents the maps of the calculated SU values. Each panel in the image shows a different agent training method. The horizontal axis of each map displays the positions of the message(steps), and the vertical axis of each map represents the input attributes. SU distributions of the baseline and weight decay approaches (Fig 12A and 12B) contain mostly weak connections, except in a few instances under weight decay. Furthermore, the SU distributions in most maps relating to weight decay and baseline approaches are uniform, implying limited specificity in connecting attributes to positions in the message. Agents when not influenced by functional pressures, distribute attribute information along the entire message, causing entanglement and complexity.

However, when agents are trained with either of the functional pressures, there is only a certain set of (*a*, *t*) pairs that contain high SU. Such an observation implies that agents learn to connect attributes to specific positions in messages, yielding better positional disentanglements. Furthermore, there is at least one position in the messages where a given attribute gets strongly connected. The SU values of such connections are high, with the lowest recorded value for any functional pressures being 0.87. Such high specificity is rarely seen in baseline methods and weight decay.

Fig 12D shows several duplicated connections with lower SU for grounding sparsity. Map of *M*(10, 8) in Fig 12D depicts that multiple positions of a message get connected with a single attribute. For example, attribute 3 share it’s information between 3^*rd*^, 4^*th*^ and 5^*th*^ positions of a message. However, later in this section, we explain this behavior further and show that sparse grounding pressure induces disentanglement differently.

We observe that agents do not necessarily mirror the order of attributes in the messages. For example, the first attribute can be mapped into the last position in messages. Nonetheless, both functional pressures tend to produce high-strength connections along the diagonal of the map, suggesting that agents tend to connect attributes in order or reverse order. We do not investigate the reasons behind this observation.

Agents develop strong but duplicated connections when training with topological similarity. In Fig 12C, *M*(10, 8) every attribute gets connected to two consecutive positions in the messages. However, such connections are still strong as indicated by high SU, and the attribute information is still connected to specific positions, unlike in weight decay and baseline. Although some redundancy is present, topological similarity causes the most orderly groundings between all the approaches, further validating our intuition in Section 3.3.

**Fig 13:**
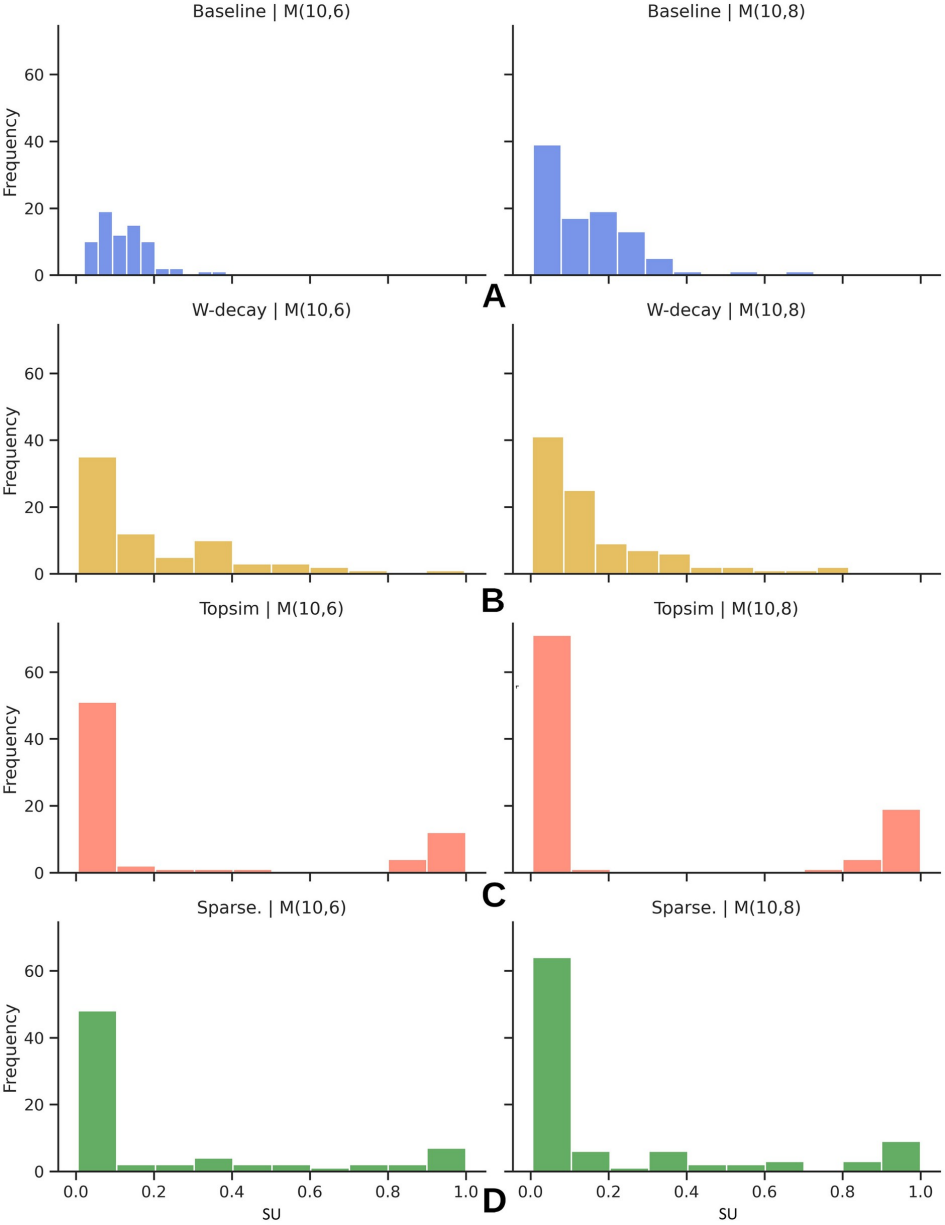
Histogram of the symmetric uncertainity between attributes and message positions for each method at different channel configurations. Each panel displays the histogram of the linkage strength, quantified by SU, for a different channel configuration. Sparse correlation and topological similarity yield histograms with two distinct peaks at the extremes. A: Baseline. B: W-decay. C: Topological similarity D: Sparse grounding.

We then plot the histogram of the strength of all such connections in Fig 13. Since our inputs originate from value attribute datasets, an optimal histogram depicting linking strength should contain only two distinct peaks at zero and one, implying no connections with intermediate SU. Each attribute should establish an unambiguous connection with a specific position within the message. However, histograms for baseline and weight decay display a pattern where we observe plenty of connections with intermediate SU. Contrarily, training with topological similarity and sparse groundings causes the SU values to concentrate around the extremes.

**Fig 14:**
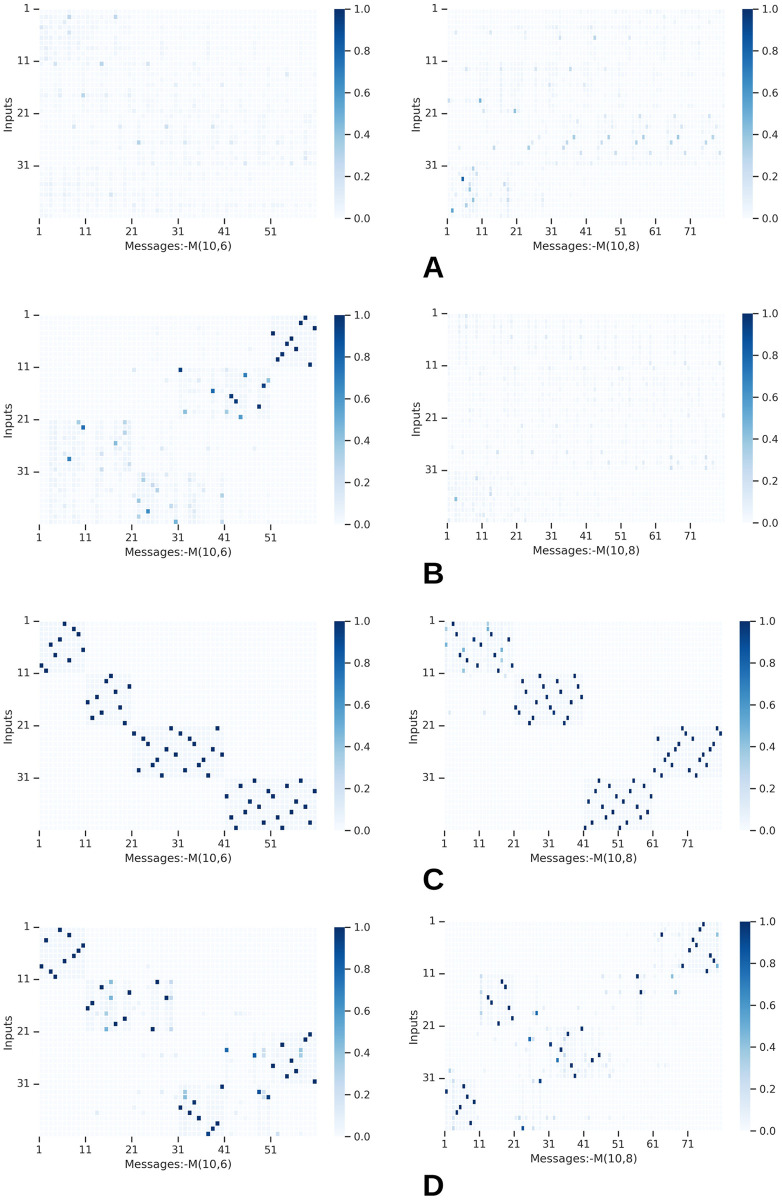
Value to symbol linkage approximated by symmetric uncertainity across all attributes and message positions. Each cell in a panel displays how much a value is connected to a symbol. In topological similarity, we observe a single value being mapped into multiple symbols, although in a controlled, structured manner. The redundant value to symbol mappings is lower when agents train with sparse grounding auxiliary loss. A: Baseline. B: W-decay. C: Topological similarity. D: Sparse grounding.

We then investigate how a given value is connected with a symbol in the messages by plotting SU(*v*_*a*_, *s*_*t*_) in Fig 14. We observe functional pressures causing better disentanglement in groundings between values and symbols. Fig 12C demonstrates how a single attribute is mapped into multiple positions of a message when topological similarity is employed. In Fig 14C, too, we can observe agents mapping a single value to two symbols at two different positions in the message, further cementing the notion that topological similarity causes some redundant connections in the grounding maps. Observed redundancy appears at both attribute-position and value-symbol levels. Nonetheless, such connections are not weak or thinly distributed similar to what is observed when functional pressures are not employed.

In Fig 12D, we observe certain low-intensity and redundant connections for grounding sparsity. However, Fig 14D shows that the number of redundant connections is much lower than other approaches if connections between individual values and symbols are taken into account. The few redundant (*a*, *t*) linkages in Fig 12D, characterized by lower symmetric uncertainty, stem from agents connecting a few values belonging to a single attribute across multiple positions within the messages. Nevertheless, agents mostly dedicate only a single symbol for a single value. This behavior is different from the situation for topological similarity, where a single value is connected to multiple symbols. Hence, when agents are constrained by the grounding sparsity pressure, the redundancy is not observed at the value to symbol level. Such observations highlight the qualitative differences in languages caused by the functional pressures.

### 5.2 Probabilistic context-free grammar

Next, we delve into analyzing our languages using unsupervised grammar induction techniques. They offer insight into the syntax of the language. It is relevant for both the researchers who are interested in syntax and also in semantics from a compositional point of view [18]. We use the CCL-BMM technique in accordance with [18]. They propose a two-stage grammar induction process in which the unlabelled constituency structures are inferred and labeled. The labeled structures are used to create probabilistic context-free grammar (PCFG).

In our comparisons, we compare syntactical properties of the languages developed by applying functional pressures with three other emergent languages :- 1. The baseline, 2. A language developed with weight decay and 3. with a structured baseline. We use the same structured baseline which is used by [18]. It is a language that is similar in length and has the same vocabulary size, but its grammar is generated from a context-free grammar with a basic hierarchy and terminal-class structures. However, we do not use the random and shuffled baselines as the previous work do, because our intention is to compare our methods against the existing language emergence methods.

In our visual analysis we investigated the languages emerged under channel configurations *M*(10, 6) and *M*(10, 8). For channel configurations with larger vocabularies, visualizing the relationship between each and every value and symbol leads to complex maps, which are harder to analyse visually. Hence, we kept such channel configurations to be analyzed with CCL-BMM. For analysis with CCL-BMM we use the messages emitted by agents training on a (10, 4) dataset with two channel configurations (100, 6) and (100, 8). We first measure the Grammar Description Length (GDL) and Data Description Length (DDL) of the emerged languages. GDL represent the number of bits needed to encode the grammar, while the code length needed to describe the data given the model is represented by the DDL.

Table 9 shows the data and grammar description lengths for 4 emergent languages with the structured baseline. It is clear that our values are still far behind the structured baseline, but this is expected. However, we observe that GDL and DDL values are lower when agents are trained with topological similarity loss or sparse grounding loss. This observation proves the grammars that is associated with our approaches are more compressible. Noticeably, at *M*(100, 8) sparse grounding method achieves nearly a 10 times lower GDL while keeping the DDL also at a lower level.

**Table 9:** GDL and DDL of emergent languages.

Channel	Method	GDL (k)	DDL
*M*(100, 6)	Baseline	34.26 ± 38.27	35.73 ± 5.88
	W-decay	23.92 ± 18.25	35.00 ± 5.07
	Topsim	10.49 ± 69.48	32.92 ± 6.87
	Sparse	6.93 ± 2.88	25.14 ± 4.79
	Structured	0.57 ± 0.00	17.93 ± 0.08
*M*(100, 8)	Baseline	122.45 ± 69.76	34.82 ± 11.92
	W-decay	95.33 ± 79.18	36.33 ± 13.17
	Topsim	20.71 ± 14.46	27.58 ± 0.91
	Sparse	10.49 ± 10.25	24.20 ± 2.61
	Structured	0.454 ± 0.00	18.01 ± 0.00

Languages emerged with our functional pressures report less GDL and DDL than the other emergent languages.

Next, we show the number of unique pre-terminal groups in Table 10. The number of pre-terminal groups detects whether there is a linguistic structure that prescribes which terminals should belong together. If there is such structure, the language can be explained with a fewer number of groups [18]. We observe that our languages have fewer unique pre-terminal groups than other emergent languages. The lowest number is recorded for the structured baseline, but this is expected, like in the prior case that concerned GDL and DDL.

**Table 10:** Number of unique pre-terminal groups.

Channel	Method	Number of unique pre-terminal groups
*M*(100, 6)	Baseline	140.83 ± 179.24
	W-decay	96.33 ± 72.79
	Topsim	78.66 ± 44.83
	Sparse	68.5 ± 34.66
	Structured	3.00 ± 0.00
*M*(100, 8)	Baseline	288.50 ± 253.64
	W-decay	196.50 ± 164.61
	Topsim	117.50 ± 59.74
	Sparse	79.66 ± 78.06
	Structured	1.00 ± 0.00

Languages emerged with our functional pressures report less number of unique pre-terminal groups

We also notice that our languages can be defined with a lower number of production rules. Such an observation should explain the faster convergence rates observed in prior experiments. Even when the channel capacities are large, which enable every method to reach perfect test accuracy, our functional pressures provide enhanced learning speeds as the underlying language they learn is composed of a simpler set of rules.

## 6. Discussion and conclusions

Our experimental results outline multiple benefits of applying topological similarity and grounding sparsity as auxiliary losses in language emergence, including enhanced generalization, rapid learning, resilience to low training data, and robustness for low-capacity communication channels. Both functional pressures yield more simple and structured emergent languages than the existing methods. Among the two functional pressures, the sparsification of groundings produces the most favorable results, and each develops languages with unique characteristics. Presented auxiliary losses are versatile and applicable under various value-attribute environments, batch sizes, and learning rates. Furthermore, deploying our approaches is straightforward, as it does not require agent replacement or multiple generations of agents.

Topological similarity is not a novel notion in emergent communication studies. The idea that compositionality can be measured through topological similarity has been posited, with methods such as neural iterated learning aiming to amplify it within agent languages. Nevertheless, its use as a differentiable auxiliary loss has not been investigated before this work, which we do for enhancing compositionality. Sparse groundings, which we also present as a differentiable auxiliary loss, is a conceptually novel idea not previously presented in language emergence literature.

Increasing the generalization and learning speed while reducing the dependency on large amounts of training data will help move language emergence research toward more practical applications. In certain learning environments, the availability of training data may be limited. For example, in situations requiring physical interaction, obtaining a large number of training trials covering every condition is not feasible. Additionally, when a large population of agents needs to coordinate with each other, it is beneficial for them to transfer and learn communications as fast as possible. Such applications benefit by methods that expedite the learning process.

Our study uses Pearson correlation to measure the strength of links between concepts and messages when formulating the auxiliary losses. However, mutual information should be a more suitable measure than Pearson correlation, as it captures non-linear and non-monotonic relationships. In this study, we focused on two novel functional pressures related to compositional structure and sparsity, proving the fruitfulness of searching functional pressures other than the least effort pressure. Further experiments that involve a combination of different functional pressures should yield exciting results and provide a more accurate understanding of the potential for large-scale applications. These experiments are left as future work.

## Supporting information

S1 Appendix Reconstruction game. (PDF)

S2 Appendix Agent architecture. (PDF)
