# Supplementary material for: Accelerating language emergence by functional pressures
Source: PLoS One. 2023 Dec 14;18(12):e0295748. doi: 10.1371/journal.pone.0295748 (PMC10721031; doi:10.1371/journal.pone.0295748)
Supplement: S1 Appendix — (PDF) [file pone.0295748.s001.pdf]

S1 Appendix. Reconstruction Game

Our variant of Lewis signalling game can be explained by the real world analogy *"Draw my picture"* game. Such simple cases, which are also used in similar studies [1, 2], are easy to implement and analyse. In our experiments, there are two players namely, Speaker and Listener. First the Speaker receives an image chosen at random from a set of images. Then he describes it to the Listener, who should draw a picture depicting the Speaker's image. Both players are rewarded if the original image and the picture drawn by the Listener are similar. Since the Listener tries to reconstruct the original image of the Speaker, this type of game is also known as a *"Reconstruction Game"*.

In our experiments, we model the players using two neural networks, which are connected by a discrete communication link. We refer to these two neural networks as agents. Instead of a real image, the Speaker receives an object sampled from a value-attribute environment. Moreover, no physical sketching is required; the Listener simply predicts the value of each attribute. Both agents are rewarded in proportion to how closely their predictions match the original values.

When converting the aforementioned situation to a language emergence experiment, an important restriction is that agents have no access to previously acquired linguistic knowledge. The Speaker is capable of emitting discrete messages, which are heard by the Listener. However, neither agents initially understand the meaning of these messages. They should gradually learn to link concepts within their environment to the messages, with the help of the reward signal.

Speaker, as defined in Equation 1, maps the inputs $\mathcal{X}$ to the set of messages $\mathcal{M}$. After observing an object $x \in \mathcal{X}$, the Speaker emits a message $m \in \mathcal{M}$. Listener, who reads the message (see Equation 2), predicts the values of the attributes of the original input. These predictions make for an approximation $x\prime$ of the original input. Reconstruction loss $\mathcal{L}$ is the cross entropy loss between the original input x and the approximation $x\prime$ (refer Equation 3).

$$Speaker : \mathcal{X} \longrightarrow \mathcal{M} \tag{1}$$

$$Listener : \mathcal{M} \longrightarrow \mathcal{X} \tag{2}$$

$$\mathcal{L}_{reconstruction} = \mathcal{L}(x, x\prime) \tag{3}$$

Discriminative games are an alternative to the reconstruction games. In a discriminative game, Speaker receives a reference object or a image similar to a reconstruction game. Listener receives a set of objects or images, which contain a copy of the reference and a set of distractors. After listening to the Speaker's messages, Listener has to identify the reference object apart from the distractors. Agents trained in discriminative settings, may communicate using a lexicon associated with low-level features of the images [3]. For instance, agents could tie their messages to the average pixel intensity of the images rather than connecting to higher-level features such as image class [4]. In a value attribute environment this could be the average of the values in the input. Then the Listener can pickup the reference easily by considering the difference of the average values across the set of objects. To prevent the emergence of such degenerative communication protocols, our experiments employ a reconstruction setting. In a reconstruction game, the Listener must precisely rebuild the Speaker's object. Hence, unlike in a discriminative setting, degenerative protocols will not emerge.

References

1. Resnick C, Gupta A, Foerster J, Dai AM, Cho K. Capacity, Bandwidth, and Compositionality in Emergent Language Learning. In: Proceedings of the 19th International Conference on Autonomous Agents and MultiAgent Systems; 2020. p. 1125–1133.

2. Gupta A, Resnick C, Foerster J, Dai A, Cho K. Compositionality and Capacity in Emergent Languages. In: Proceedings of the 5th Workshop on Representation Learning for NLP; 2020. p. 34–38.

3. Bouchacourt D, Baroni M. How agents see things: On visual representations in an emergent language game. In: Proceedings of the 2018 Conference on Empirical Methods in Natural Language Processing; 2018. p. 981–985.

4. Kharitonov E, Chaabouni R, Bouchacourt D, Baroni M. Entropy minimization in emergent languages. In: International Conference on Machine Learning. PMLR; 2020. p. 5220–5230.
