# Supplementary material for: Accelerating language emergence by functional pressures
Source: PLoS One. 2023 Dec 14;18(12):e0295748. doi: 10.1371/journal.pone.0295748 (PMC10721031; doi:10.1371/journal.pone.0295748)
Supplement: S2 Appendix — (PDF) [file pone.0295748.s002.pdf]

S2 Appendix. Agent architecture

Fig 1 shows the architecture of the Speaker and the Listener. Agents are modeled as recurrent neural networks using LSTMs [1], as it is frequently used in language emergence literature [2–6]. First a one-hot encoded input x from a value-attribute environment is fed into the speaker's LSTM cell via a single feed-forward layer $FC1$ and a batch normalization layer BN as both the initial cell state c_0 and hidden state h_0. The initial input s_0 is initialized to zeros and is fed to the LSTM cell via a set of Embeddings.

The output hidden state h_1 of the LSTM cell is forwarded through the linear layer $FC2$ to sample a symbol $s_1 \in S$ using Gumbel-softmax sampling [7]. As the input for the next time step, the sampled symbol s_1 from the current step is fed back to the LSTM cell through the same set of embeddings. This process is repeated until the number of sampled symbols are equal to the message length T. After reaching the message length, the sampling process is stopped, and the message $m \in \mathcal{M}$ formed by concatenating the discrete symbols $s_1, \ldots, s_T$, is forwarded to the Listener.

An LSTM layer inside the Listener receives the message first and consume it entirely, and its hidden state is fed to a single linear layer $FC3$. The output un-normalized log probabilities of $FC3$ are considered as a representation of the reconstruction x' and it is used with the cross entropy to calculate the reconstruction loss.

In all our experiments, we maintain a constant hidden state size of 500 for both the Speaker and the Listener. However, the dimensions of the fully connected layers are varying to accommodate different value-attribute datasets and channel capacities. $FC1 : |x| \longrightarrow 500, FC2 : 500 \longrightarrow S, FC3 : 500 \longrightarrow |x|$

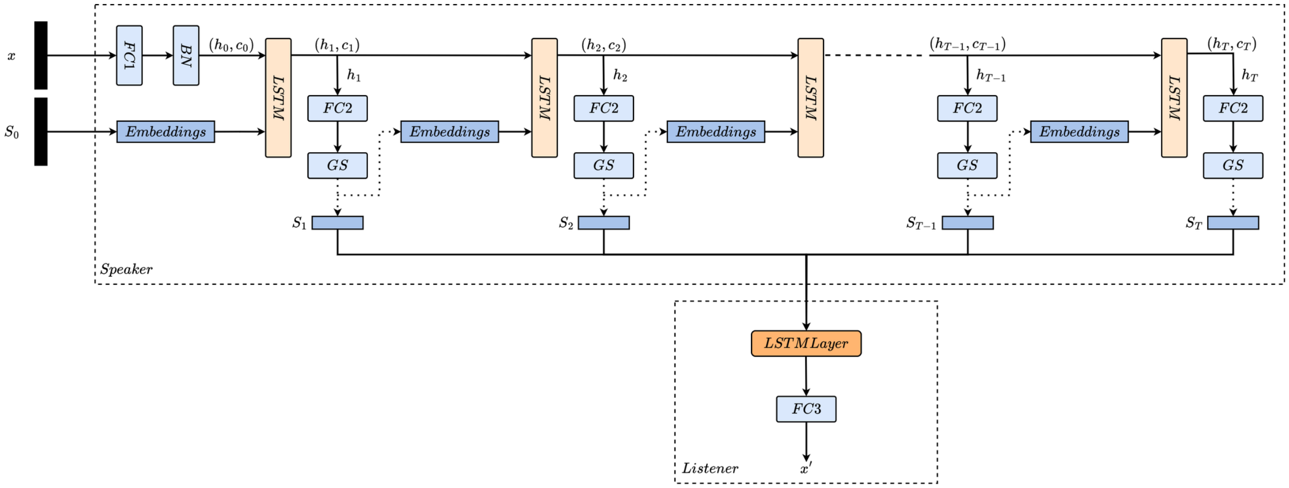

Fig 1. Model architecture of the Speaker and the Listener, engaged in a reconstruction game.

We use a linear agent architecture for the experiments in Experiment set 5 to inspect whether our functional pressures perform adequately with non-recurrent agents. In this experiment, we model each agent with 2 linear layers. Sender ingests the entire input x with its first linear layer FC_{s1}, and its outputs are forwarded to the second linear layer FC_{s2}. The output of the second layer of the Sender is used to sample the message. Listener ingests the incoming message with its first linear layer FC_{l1} and the output is fed to the second linear layer FC_{l2}, which produces un-normalized log probabilities representing the reconstruction x'.
$FC_{s1} : |x| \longrightarrow 2|x|, FC_{s2} : 2|x| \longrightarrow ST, FC_{l1} : ST \longrightarrow 2|x|, FC_{l2} : 2|x| \longrightarrow |x|$.

References

1. Hochreiter S, Schmidhuber J. Long short-term memory. Neural computation. 1997;9(8):1735–1780.

2. Evtimova K, Drozdov A, Kiela D, Cho K. Emergent communication in a multi-modal, multi-step referential game. In: 6th International Conference on Learning Representations; 2018.

3. Havrylov S, Titov I. Emergence of language with multi-agent games: Learning to communicate with sequences of symbols. In: Advances in neural information processing systems; 2017. p. 2149–2159.

4. Ren Y, Guo S, Labeau M, Cohen SB, Kirby S. Compositional languages emerge in a neural iterated learning model. In: International Conference on Learning Representations; 2020.

5. Mu J, Goodman N. Emergent Communication of Generalizations. Advances in Neural Information Processing Systems. 2021;34.

6. Liang PP, Chen J, Salakhutdinov R, Morency LP, Kottur S. On emergent communication in competitive multi-agent teams. arXiv preprint arXiv:200301848. 2020;.

7. Jang E, Gu S, Poole B. Categorical Reparameterization with Gumbel-Softmax. In: 5th International Conference on Learning Representations; 2017.
